# Supplementary material for: Shared evolutionary processes shape landscapes of genomic variation in the great apes
Source: Genetics. 2024 Jan 18;226(4):iyae006. doi: 10.1093/genetics/iyae006 (PMC10990428; doi:10.1093/genetics/iyae006)
Supplement: iyae006_Supplementary_Data [file iyae006_supplementary_data.pdf]

Supplementary material

4.1 Correlation between divergences that share branches

Landscapes of divergence can be correlated by their definition, as they can share part of their histories. In most of our analyses (except for Figure S2), we do not show the correlations for such cases but below we describe how this sharing would affect correlations (using a simplified theory). For example, in Figure 3 d_{VX} and d_{XY} share the branch X; depending on how the length of the branch X compares to the total tree length, these two landscapes are bound to be correlated. Assuming that mutations follow a Poisson process and that coalescences happen instantaneously, we derive the following. There are three non-overlapping parts in the tree between these, the branch from the XY ancestor to X with length $\mathrm{E}[\tau_X] = T_{XY}$, the branch from the XY ancestor to Y with length $\mathrm{E}[\tau_Y] = T_{XY}$ and the branch from V to the XY ancestor with length $\mathrm{E}[\tau_V] = 2T_{VWXY} - T_{XY}$. If we just consider the genealogical definition of divergence and assume $d_{VX} = \tau_V + \tau_X$ and $d_{XY} = \tau_X + \tau_Y$ (i.e., ignoring the contributions of ancestral diversity to divergence), then

$$\begin{aligned}
\mathrm{Cov}[d_{VX}, d_{XY}] &= \mathrm{Cov}[\tau_X + \tau_V, \tau_X + \tau_Y] \\
&= \mathrm{Cov}[\tau_X, \tau_X] + \cancelto{0}{\mathrm{Cov}[\tau_X, \tau_Y]} + \cancelto{0}{\mathrm{Cov}[\tau_V, \tau_X]} + \cancelto{0}{\mathrm{Cov}[\tau_V, \tau_Y]} \\
&= \mathrm{Var}(\tau_X) = \mathrm{E}[\tau_X] = T_X
\end{aligned}$$

Therefore,

$$\begin{aligned}
\mathrm{Cor}[d_{VX}, d_{XY}] &= \frac{\mathrm{Cov}[\tau_X + \tau_V, \tau_X + \tau_Y]}{\sqrt{\mathrm{Var}[\tau_X + \tau_V]\,\mathrm{Var}[\tau_X + \tau_Y]}} \\
&= \sqrt{\frac{\mathrm{Var}[\tau_X]^2}{(\mathrm{Var}[\tau_X] + \mathrm{Var}[\tau_V])(\mathrm{Var}[\tau_X] + \mathrm{Var}[\tau_Y])}} \\
&= \sqrt{\frac{\mathrm{Var}[\tau_X]}{\mathrm{Var}[\tau_X] + \mathrm{Var}[\tau_V]}\frac{\mathrm{Var}[\tau_X]}{\mathrm{Var}[\tau_X] + \mathrm{Var}[\tau_Y]}} \\
&= \sqrt{\frac{T_X}{T_X + T_V}\frac{T_X}{T_X + T_Y}} \\
&= \sqrt{p_{d_{VX}} p_{d_{XY}}}
\end{aligned}$$

where $p_{d_{VX}} = \frac{T_X}{T_X + T_V}$ is the proportion of d_{VX} that is shared with d_{XY}, and $p_{d_{XY}} = \frac{T_X}{T_X + T_Y}$ is the proportion of d_{XY} that is shared with d_{VX}.

Table S1: Parameter space explored with simulations. μ_N and μ_P are the rates of mutations under negative and positive selection, respectively. $\bar{s}_N$ and $\bar{s}_P$ and the mean fitness effects of negatively and positively selected mutations. μ_{SD} is the scaled standard deviation of the mutation rate map. See Table 1 and subsection 2.2 for more details.

μ_N	μ_P	$\bar{s}_N$	$\bar{s}_P$	Regime	μ_{SD}
0	0	0	0	Neutral	0
0	0	0	0	Variable μ	0.005
0	0	0	0	Variable μ	0.007
0	0	0	0	Variable μ	0.011
0	0	0	0	Variable μ	0.016
0	0	0	0	Variable μ	0.023
0	0	0	0	Variable μ	0.033
0	0	0	0	Variable μ	0.048
0	0	0	0	Variable μ	0.070
0	0	0	0	Variable μ	0.103
0	0	0	0	Variable μ	0.150
0	1×10^{-12}	0	1×10^{-2}	Beneficial	0
0	1×10^{-11}	0	1×10^{-2}	Beneficial	0
2×10^{-9}	0	-3×10^{-2}	0	Deleterious	0
2×10^{-9}	1×10^{-11}	-3×10^{-2}	1×10^{-2}	Both	0
2×10^{-9}	0	-1.5×10^{-2}	0	Deleterious	0
2×10^{-9}	1×10^{-11}	-1.5×10^{-2}	1×10^{-2}	Both	0
2×10^{-9}	0	-1×10^{-2}	0	Deleterious	0
2×10^{-9}	1×10^{-12}	-1×10^{-2}	5×10^{-3}	Both	0
2×10^{-9}	1×10^{-12}	-1×10^{-2}	1×10^{-2}	Both	0
2×10^{-9}	0	-3×10^{-3}	0	Deleterious	0
2×10^{-9}	1×10^{-12}	-3×10^{-3}	5×10^{-3}	Both	0
2×10^{-9}	1×10^{-12}	-3×10^{-3}	1×10^{-2}	Both	0
2×10^{-9}	0	-1×10^{-3}	0	Deleterious	0
2×10^{-9}	1×10^{-11}	-1×10^{-3}	1×10^{-2}	Both	0
6×10^{-9}	0	-3×10^{-2}	0	Deleterious	0
6×10^{-9}	1×10^{-11}	-3×10^{-2}	1×10^{-2}	Both	0
6×10^{-9}	0	-1.5×10^{-2}	0	Deleterious	0
6×10^{-9}	1×10^{-11}	-1.5×10^{-2}	1×10^{-2}	Both	0
1×10^{-8}	1×10^{-11}	-1×10^{-3}	1×10^{-2}	Both	0
1.2×10^{-8}	0	-3×10^{-2}	0	Deleterious	0
1.2×10^{-8}	1×10^{-12}	-3×10^{-2}	1×10^{-2}	Both	0
1.2×10^{-8}	1×10^{-12}	-3×10^{-2}	1×10^{-2}	Both	0.005
1.2×10^{-8}	1×10^{-12}	-3×10^{-2}	1×10^{-2}	Both	0.007
1.2×10^{-8}	1×10^{-12}	-3×10^{-2}	1×10^{-2}	Both	0.011
1.2×10^{-8}	1×10^{-12}	-3×10^{-2}	1×10^{-2}	Both	0.016
1.2×10^{-8}	1×10^{-12}	-3×10^{-2}	1×10^{-2}	Both	0.023
1.2×10^{-8}	1×10^{-12}	-3×10^{-2}	1×10^{-2}	Both	0.033
1.2×10^{-8}	1×10^{-12}	-3×10^{-2}	1×10^{-2}	Both	0.048
1.2×10^{-8}	1×10^{-12}	-3×10^{-2}	1×10^{-2}	Both	0.070
1.2×10^{-8}	1×10^{-12}	-3×10^{-2}	1×10^{-2}	Both	0.103
1.2×10^{-8}	1×10^{-12}	-3×10^{-2}	1×10^{-2}	Both	0.150
1.2×10^{-8}	1×10^{-11}	-3×10^{-2}	1×10^{-2}	Both	0
1.4×10^{-8}	0	-3×10^{-2}	0	Deleterious	0
1.4×10^{-8}	0	-3×10^{-2}	0	Deleterious	0.005
1.4×10^{-8}	0	-3×10^{-2}	0	Deleterious	0.007
1.4×10^{-8}	0	-3×10^{-2}	0	Deleterious	0.011
1.4×10^{-8}	0	-3×10^{-2}	0	Deleterious	0.016
1.4×10^{-8}	0	-3×10^{-2}	0	Deleterious	0.023
1.4×10^{-8}	0	-3×10^{-2}	0	Deleterious	0.033
1.4×10^{-8}	0	-3×10^{-2}	0	Deleterious	0.048
1.4×10^{-8}	0	-3×10^{-2}	0	Deleterious	0.070
1.4×10^{-8}	0	-3×10^{-2}	0	Deleterious	0.103
1.4×10^{-8}	0	-3×10^{-2}	0	Deleterious	0.150
1.4×10^{-8}	1×10^{-12}	-3×10^{-2}	1×10^{-2}	Both	0
1.4×10^{-8}	1×10^{-11}	-3×10^{-2}	1×10^{-2}	Both	0

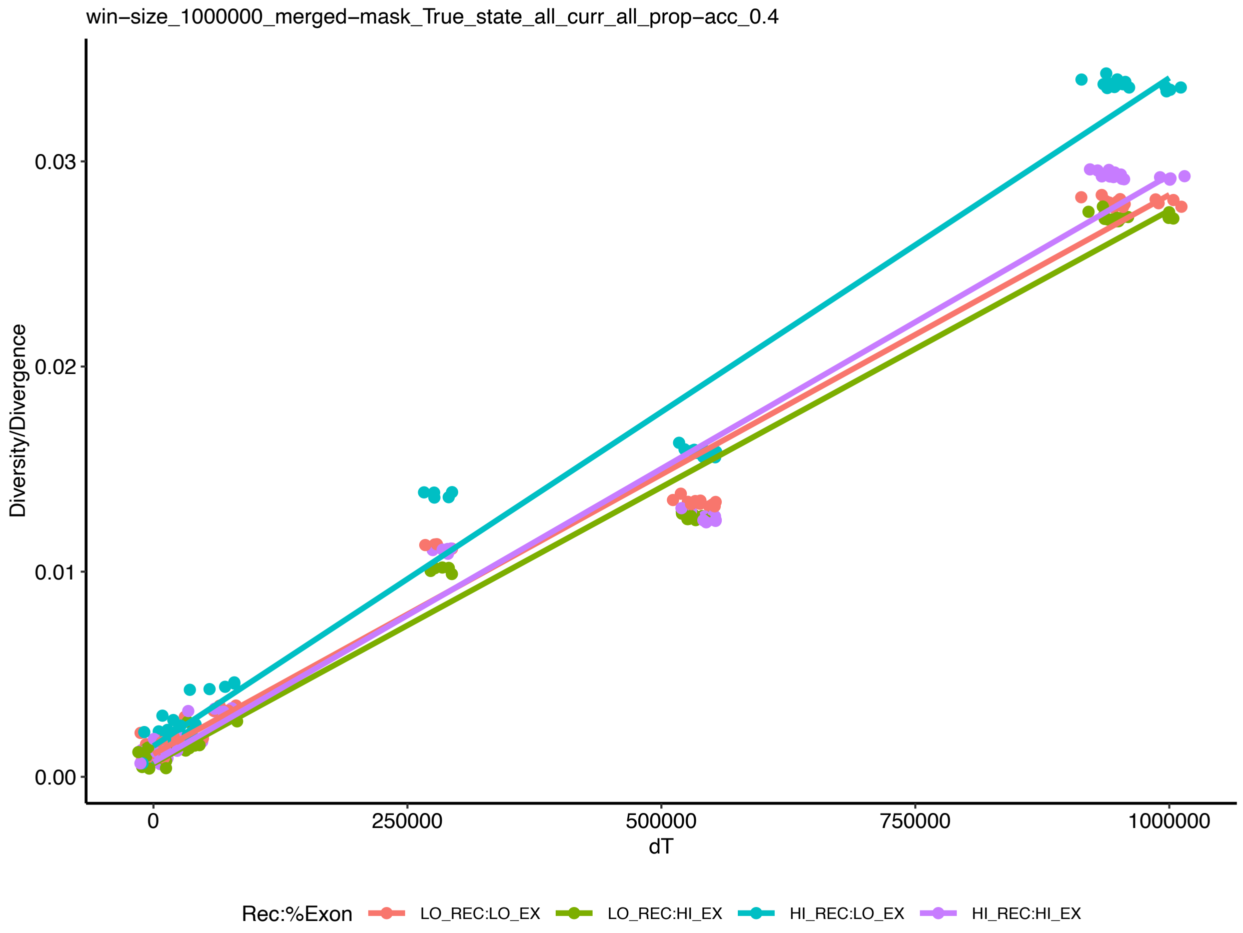

Figure S1: Effect of exon density and recombination rate on the accumulation of genetic divergence in chromosome 12 with phylogenetic distance. Within-species genetic diversities are shown at $dT = 0$. Mean diversity and divergences were computed for four groups depending on whether they fell or not on the top 90% percentile of recombination rate and exon density.

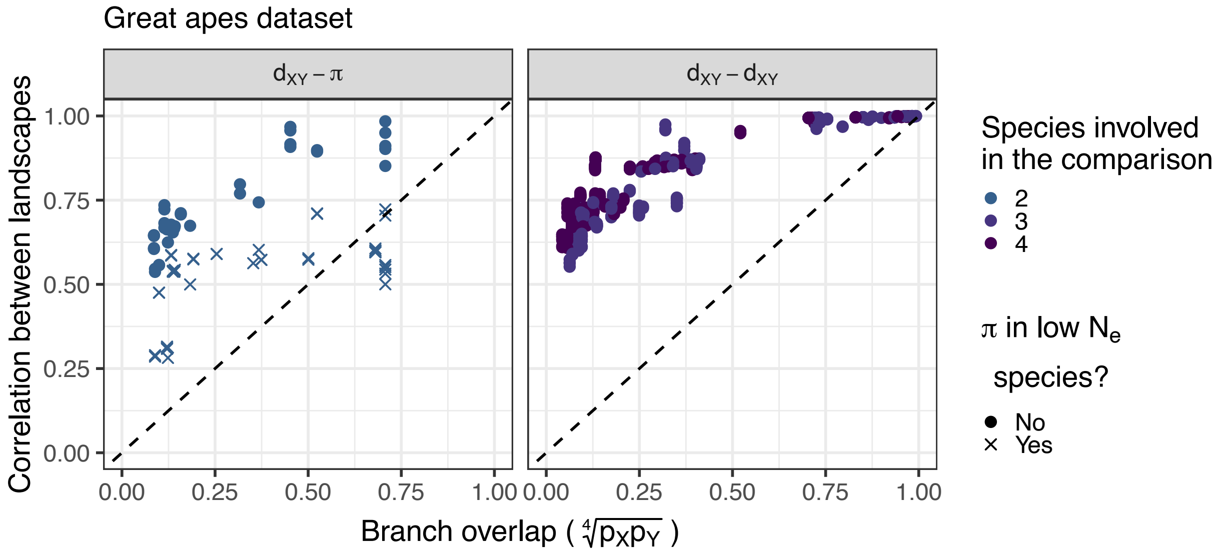

Figure S2: Correlations between landscapes of diversity and divergence for comparisons with branch overlap. For example, diversity in humans and divergence between humans and bonobos share part of their history. Each point on the plots correspond to the (Spearman) correlation between two landscapes of diversity/divergence, computed on 1Mb windows across the entire genome. Correlations were split by type of landscapes compared ($\pi - d_{XY}$, $d_{XY} - d_{XY}$). The x-axis is a metric of expected branch overlap between the landscapes. See subsection 4.1 for more information. Note that species with low N_e (bonobos, eastern gorillas and western chimps) have a different point shape. The colors reflect the number of species involved in the comparison. For example, the comparison between human-western gorilla and eastern chimp-Sumatran orangutan divergences includes four different species. On the other hand, the comparison between human-western gorilla and human-Sumatran orangutan divergences includes just three species.

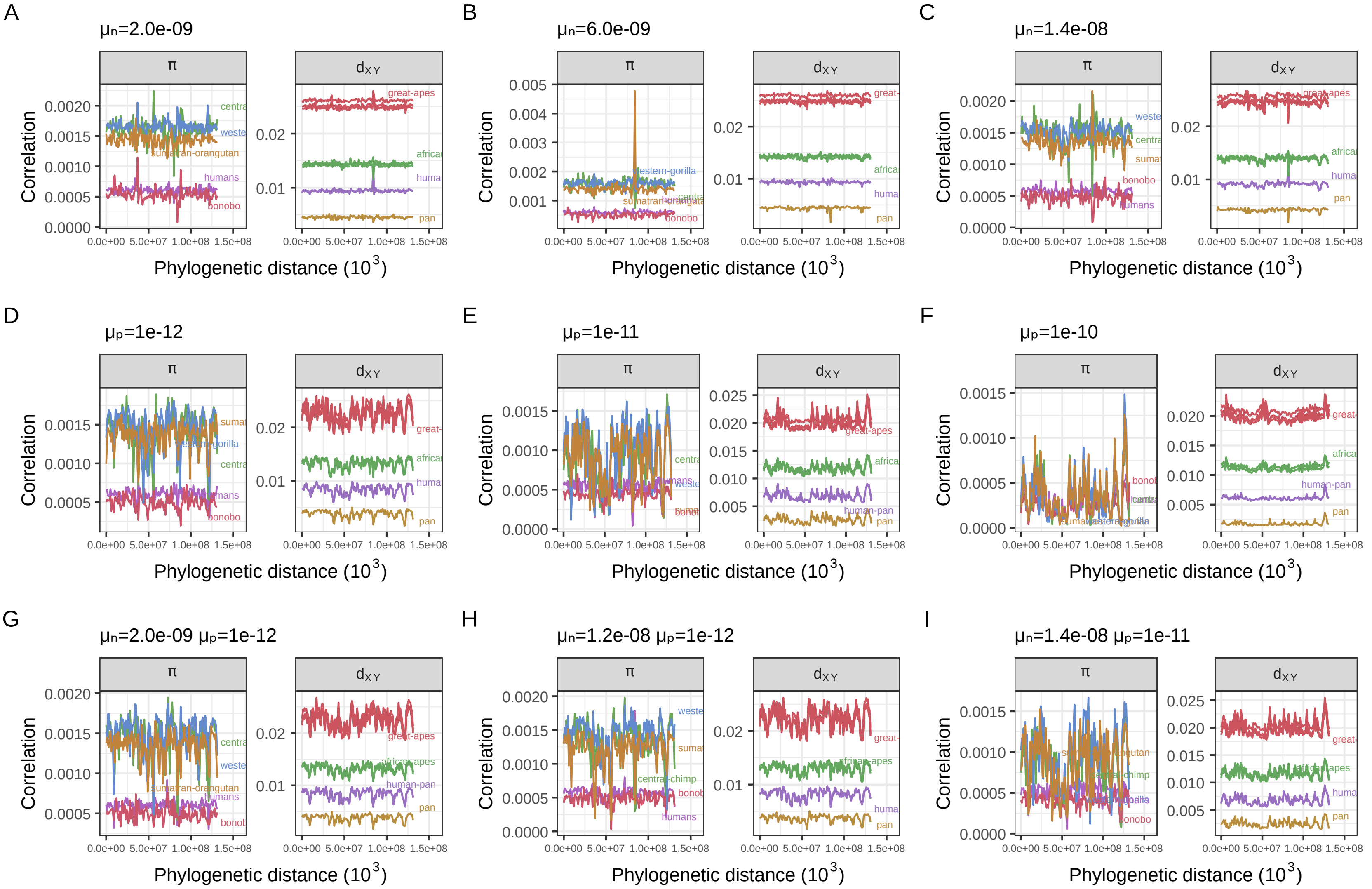

Figure S3: Landscapes of diversity and divergence in selected simulations with natural selection. The selection parameters μ_n and μ_p are the rate of mutations in exons with negative and positive fitness effects, respectively. The mean fitness effect was $\bar{s} = -0.03$ for deleterious mutations and $\bar{s} = 0.01$ for beneficial mutations (see subsection 2.2 for more details). Other details are as in Figure 2.

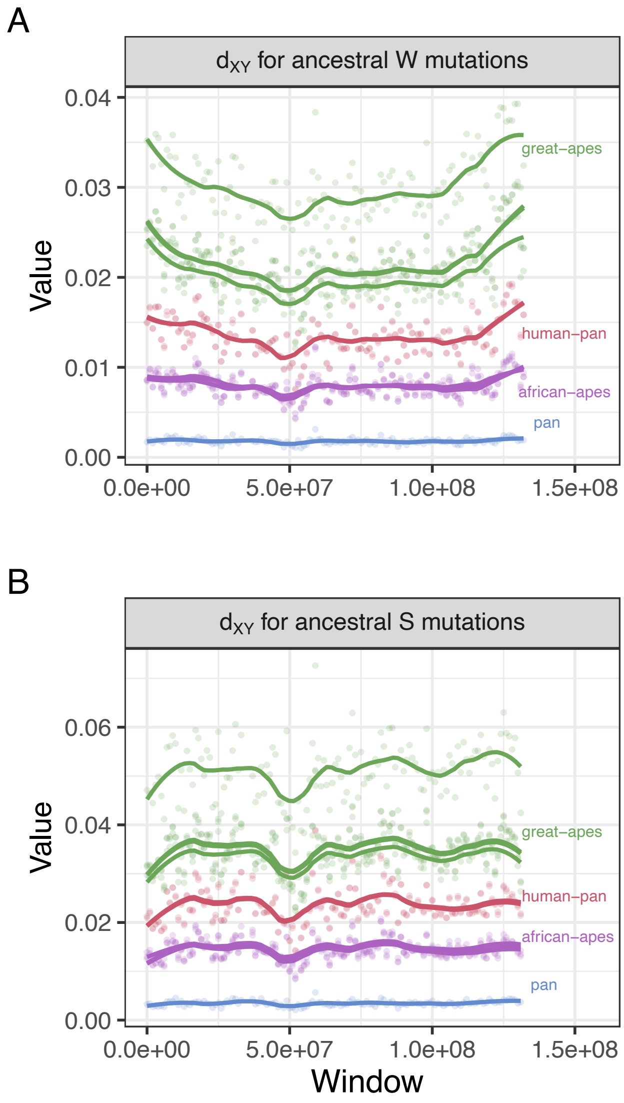

Figure S4: Landscapes of divergence partitioned by allele state in the ancestor. Ancestral states were assumed to be the same as seen in rhesus macaques (RheMac2), and sites not called in macaques were not used. d_{XY} for W sites is simply the mean pairwise differences between samples in species X and Y per ancestral W sites (A/T). Similar reasoning applies for d_{XY} for S ancestral sites, but only considering (G/C) sites. Points were colored by the most common recent ancestor of the two species compared in each divergence. Lines were fitted using local linear regression. Note that for ancestrally weak mutations (A) there is an increase in divergence at the ends of the chromosomes, but that is not seen for ancestrally strong mutations (B).

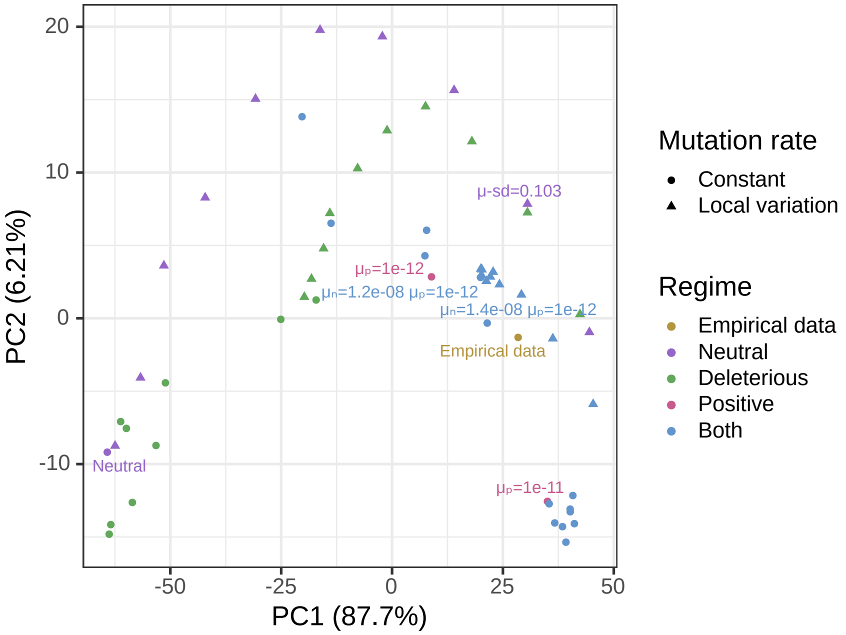

Figure S5: PCA visualization of data and simulations at 500Kb. The colors differentiate the empirical data from simulations with different parameters: Neutral refers to the simulation without any selection, Deleterious refers to simulations with deleterious mutations, Positive refers to simulations with beneficial mutations, Both refers to simulations with both beneficial and deleterious mutations. The shape of the points differentiate simulations with constant mutation rate along the genome and variable local mutation rates. Principal component analysis (PCA) applied to a matrix with all pairwise correlations between landscapes across the great apes (including $\pi - \pi$, $\pi - d_{XY}$ and $d_{XY} - d_{XY}$ comparisons) for the great apes dataset and simulations (with selection and with mutation rate variation). We excluded simulations with $\mu_p \geq 1 \times 10^{-10}$ from the PCA analysis because PC2 was capturing negative correlations caused by strong positive selection — as seen in Figure 7F.

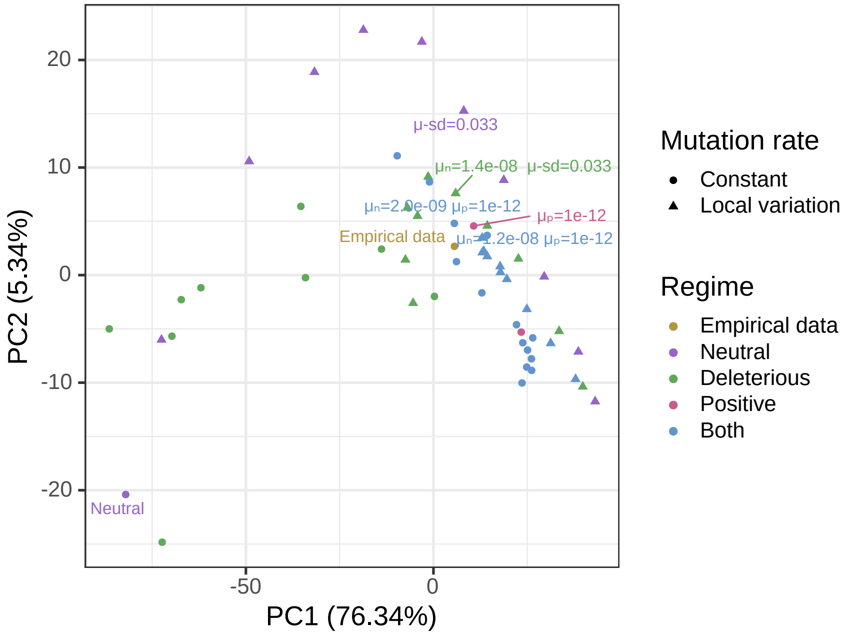

Figure S6: PCA visualization of data and simulations at 5Mb. The colors differentiate the empirical data from simulations with different parameters: Neutral refers to the simulation without any selection, Deleterious refers to simulations with deleterious mutations, Positive refers to simulations with beneficial mutations, Both refers to simulations with both beneficial and deleterious mutations. The shape of the points differentiate simulations with constant mutation rate along the genome and variable local mutation rates. Principal component analysis (PCA) applied to a matrix with all pairwise correlations between landscapes across the great apes (including $\pi - \pi$, $\pi - d_{XY}$ and $d_{XY} - d_{XY}$ comparisons) for the great apes dataset and simulations (with selection and with mutation rate variation). We excluded simulations with $\mu_p \geq 1 \times 10^{-10}$ from the PCA analysis because PC2 was capturing negative correlations caused by strong positive selection — as seen in Figure 7F.

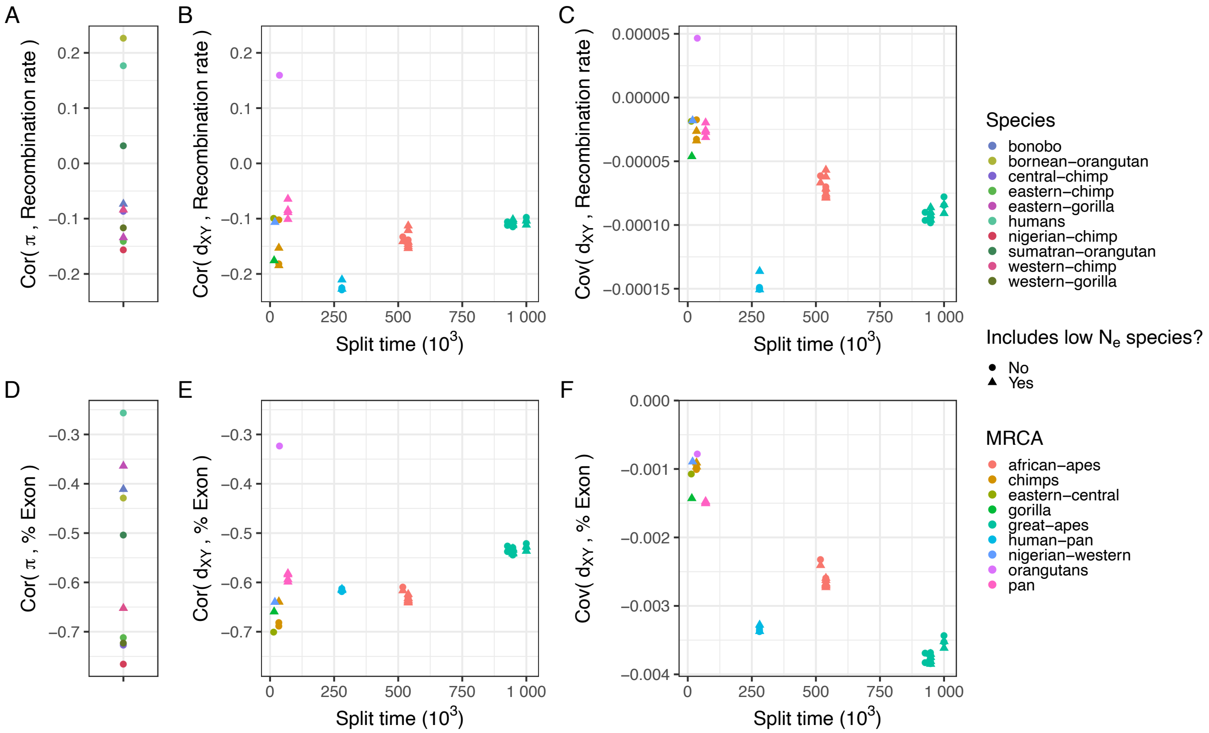

Figure S7: Correlations and covariances between landscapes of diversity and divergence and annotation features in the real great apes data. Only windows in the middle half of chromosome 12 were included. Compare to Figure 10.

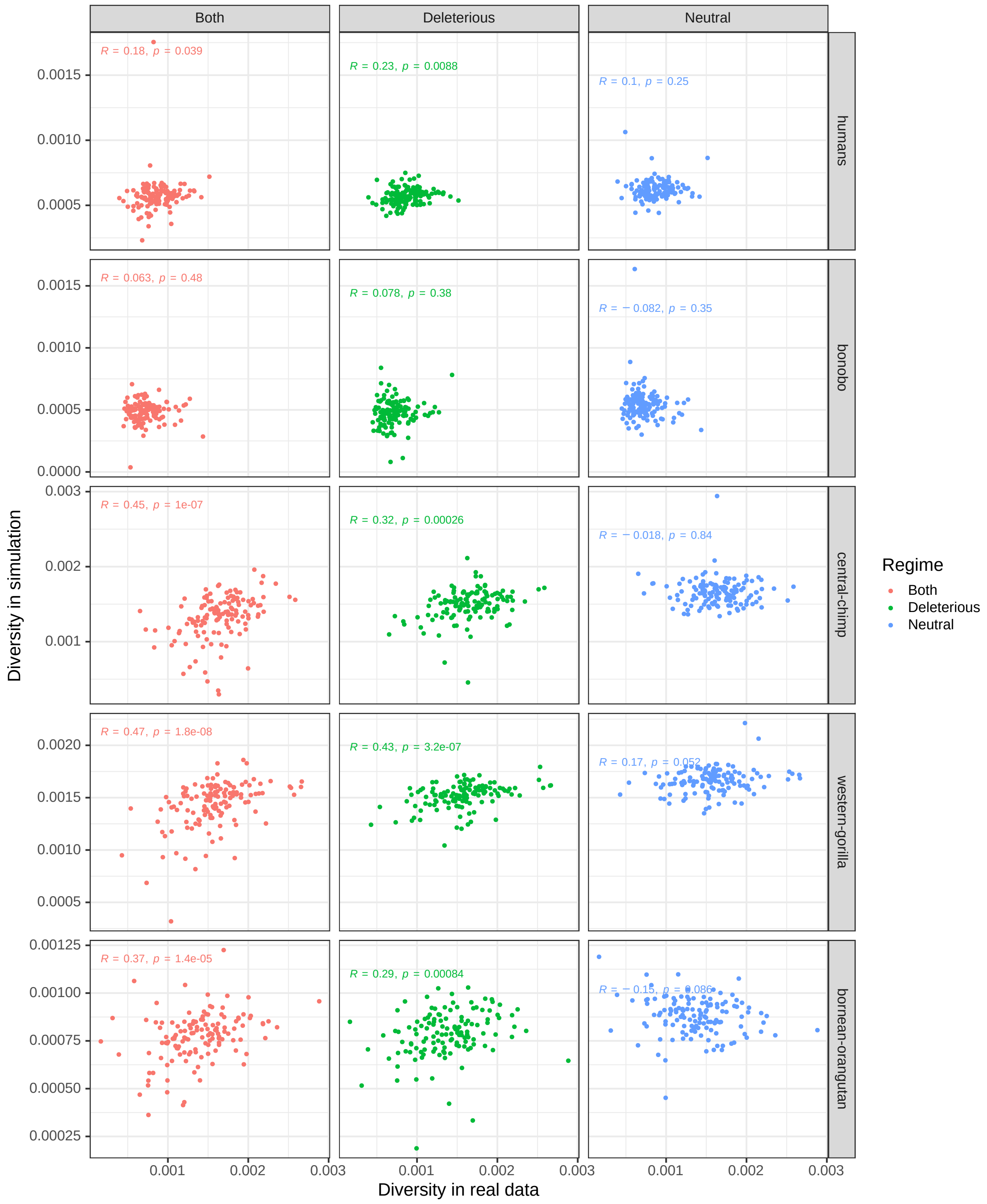

Figure S8: Scatterplots of genetic diversity in the real great apes data against diversity seen in simulations. Simulation with deleterious mutations had $\mu_n = \exp 1.4{-}8$, and the simulation with both deleterious and beneficial mutations had $\mu_n = \exp 1.4{-}8$ and $\mu_p = \exp 1{-}12$.

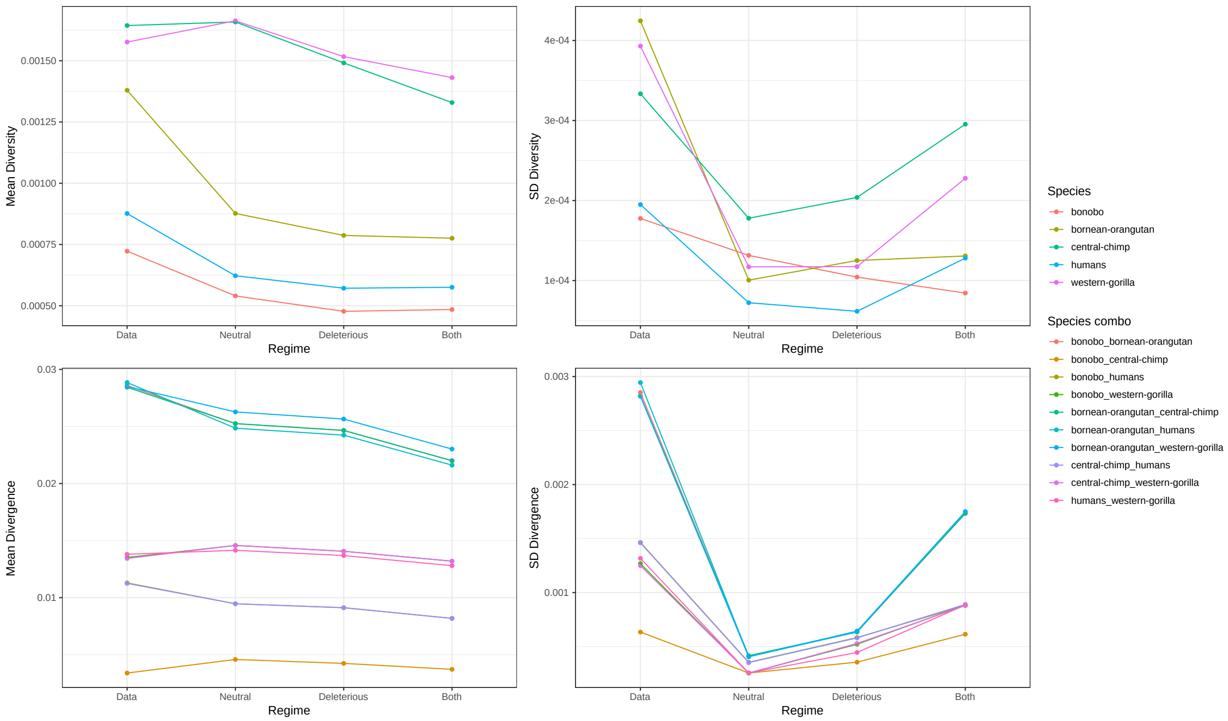

Figure S9: Summaries of genetic diversity (top) and divergence (bottom) across all species for the data and simulations. Mean is shown on the left and standard deviation on the right. "Neutral" refers to the simulation without any selection, "Deleterious" refers to the simulation with deleterious mutations ocurring at a rate of 1.4×10^{-8}, "Both" refers to the simulation with both beneficial and deleterious mutations, with rates 1×10^{-12} and 1.4×10^{-8} respectively.

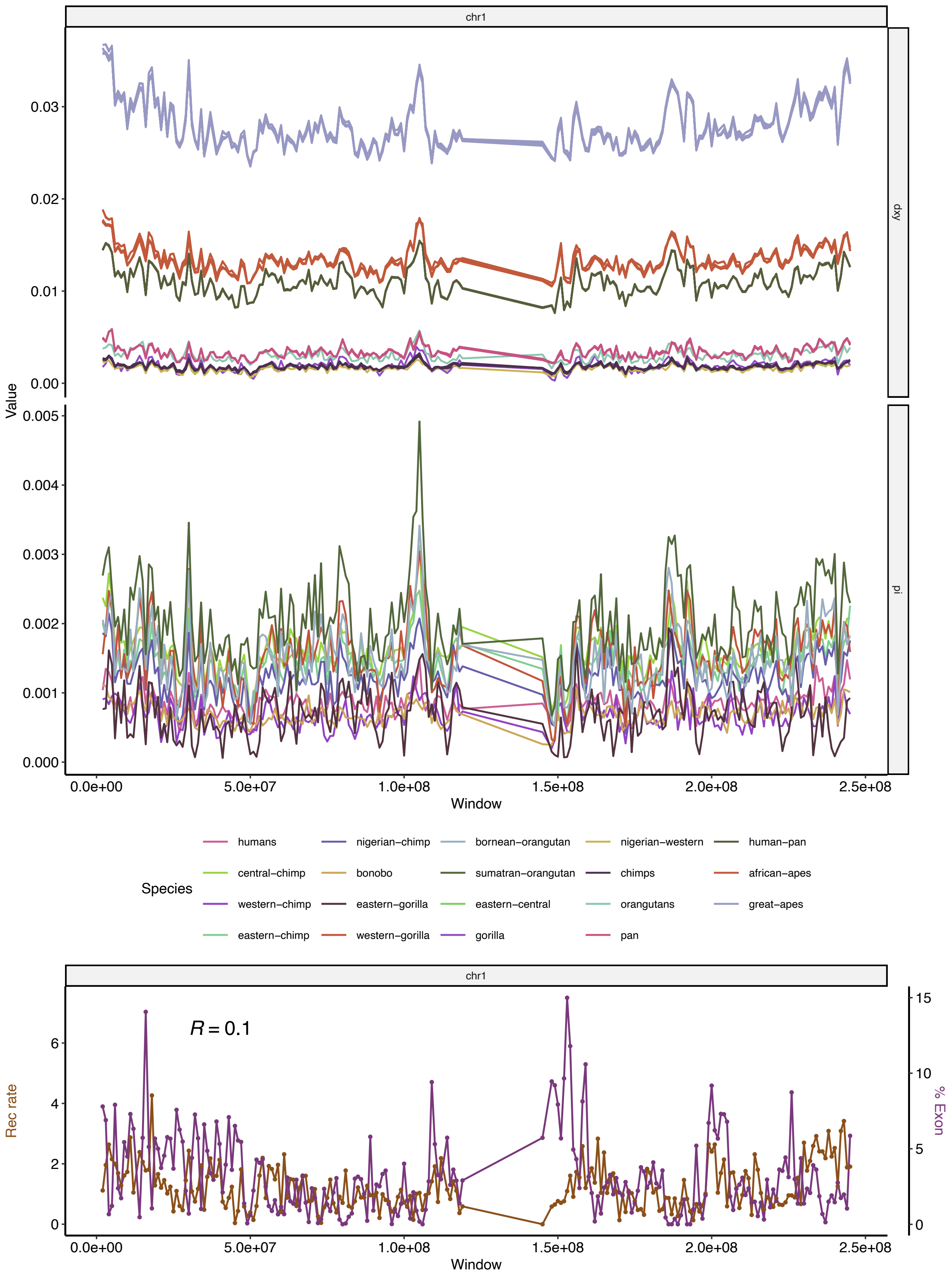

Figure S10: Landscapes of diversity, divergence, exon density and recombination rate across chromosome 1. See Figure 2 for more details.

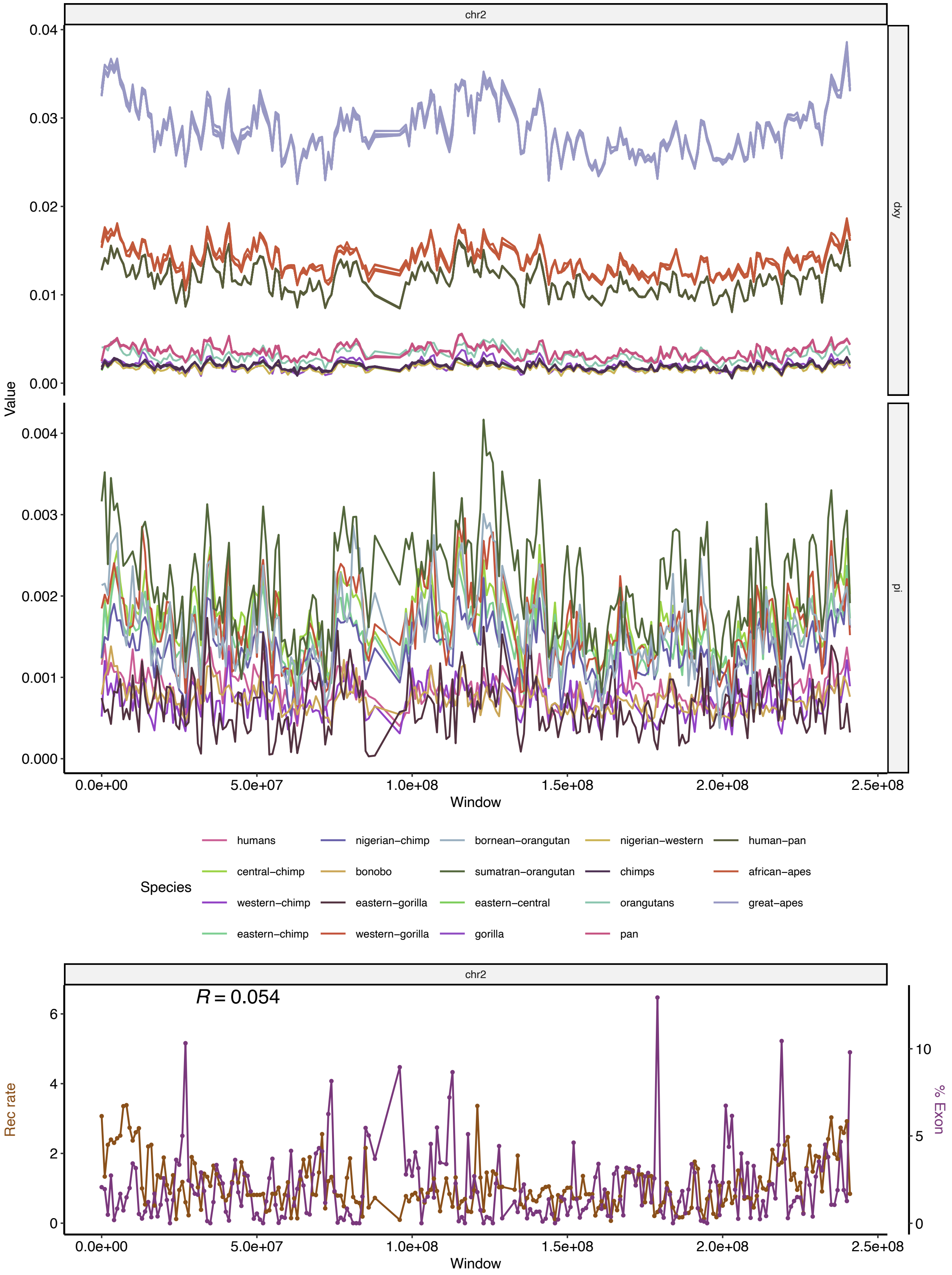

Figure S11: Landscapes of diversity, divergence, exon density and recombination rate across chromosome 2. See Figure 2 for more details.

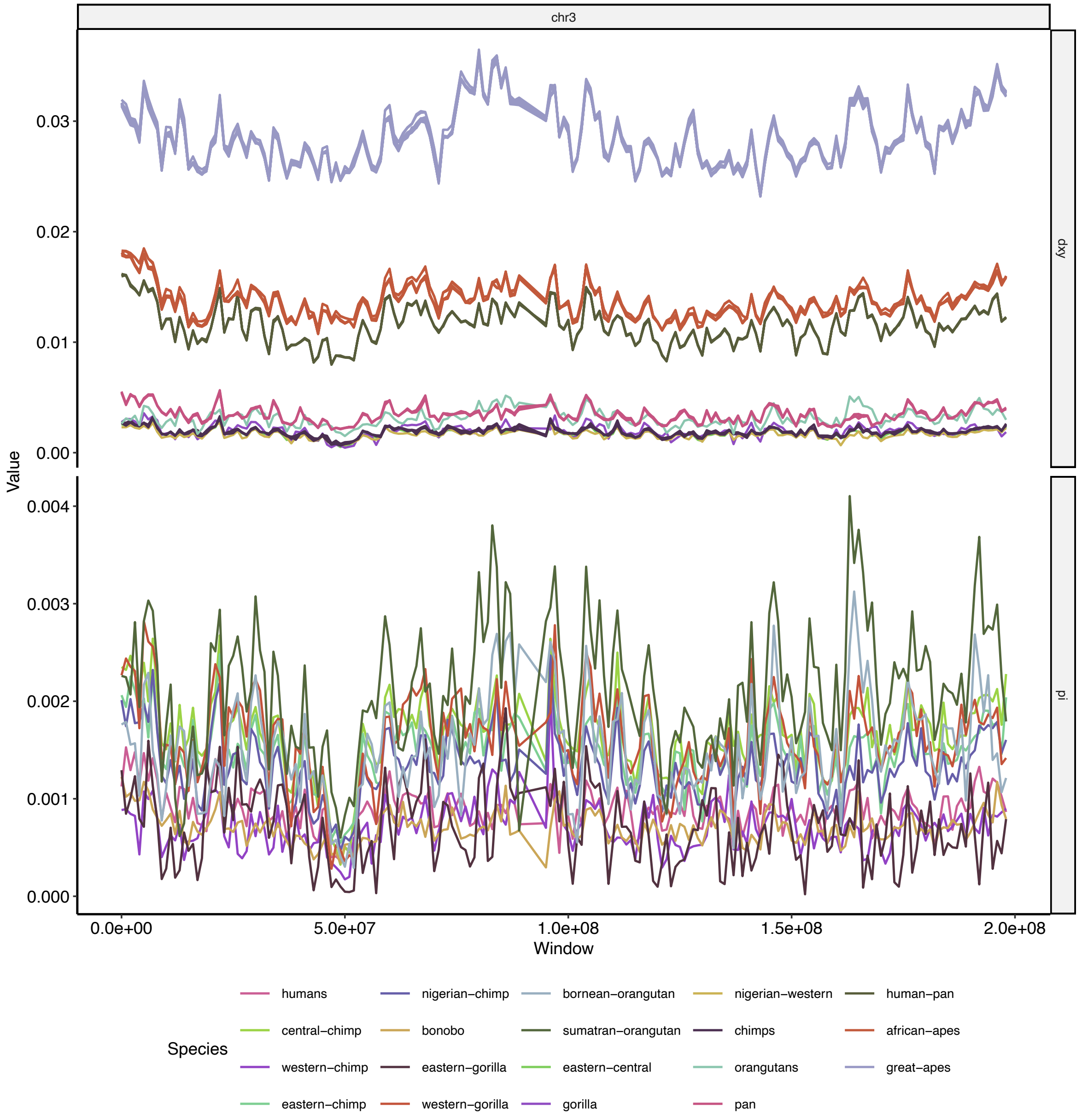

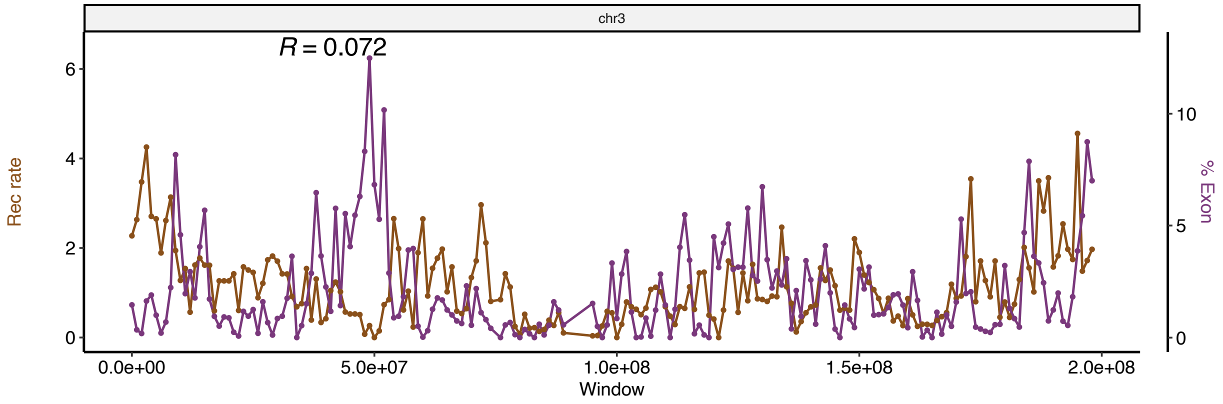

Figure S12: Landscapes of diversity, divergence, exon density and recombination rate across chromosome 3. See Figure 2 for more details.

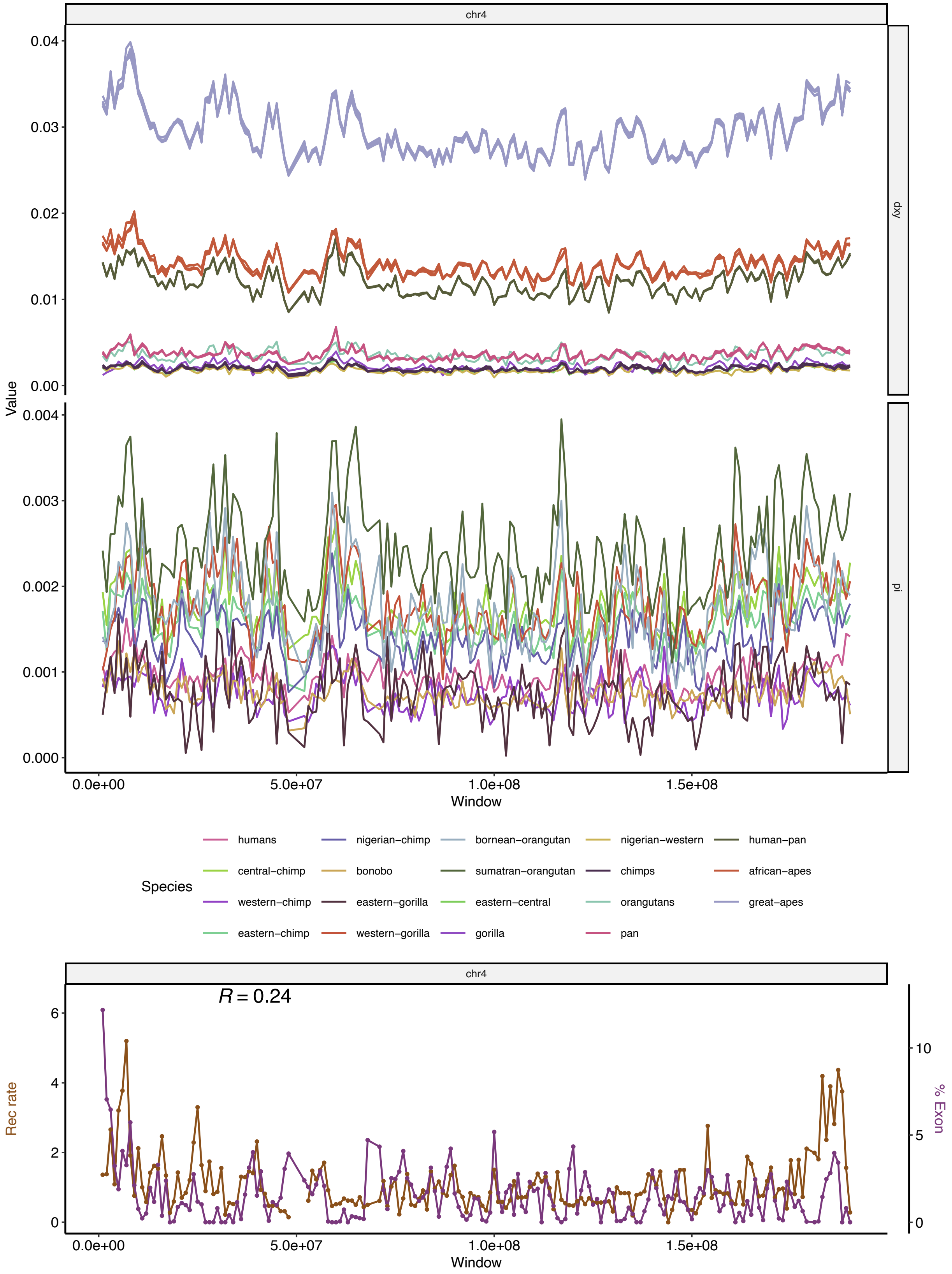

Figure S13: Landscapes of diversity, divergence, exon density and recombination rate across chromosome 4. See Figure 2 for more details.

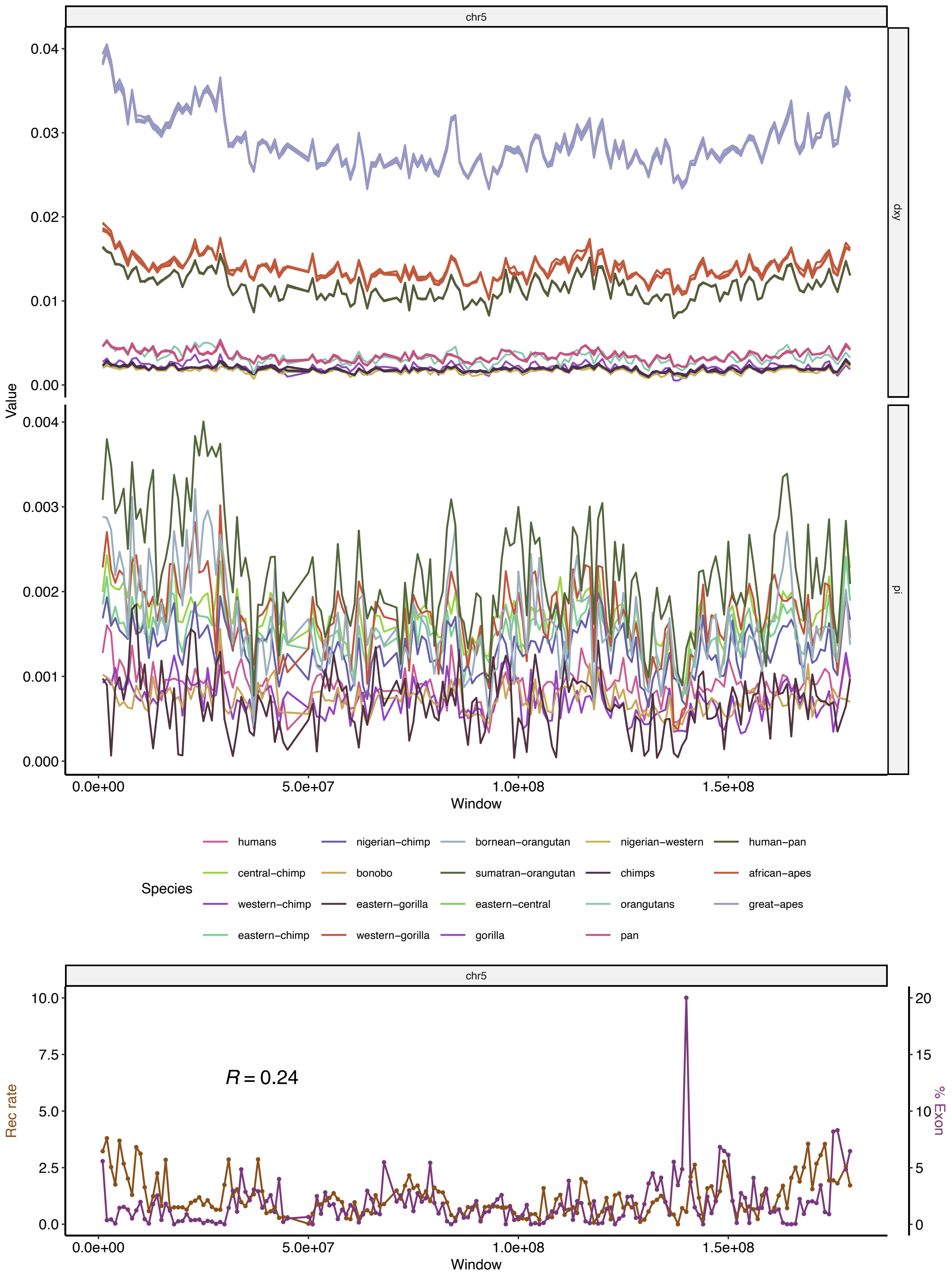

Figure S14: Landscapes of diversity, divergence, exon density and recombination rate across chromosome 5. See Figure 2 for more details.

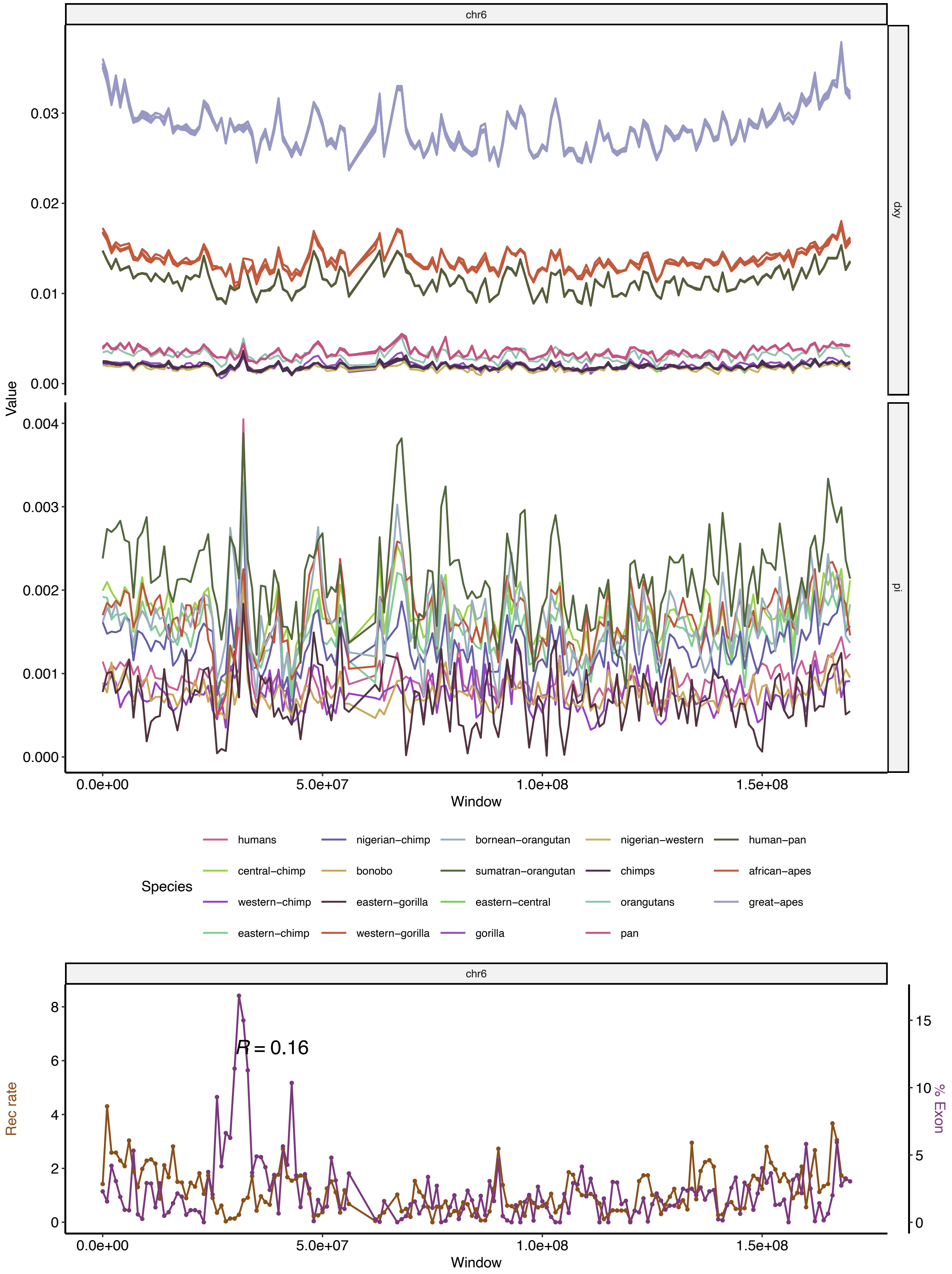

Figure S15: Landscapes of diversity, divergence, exon density and recombination rate across chromosome 6. See Figure 2 for more details.

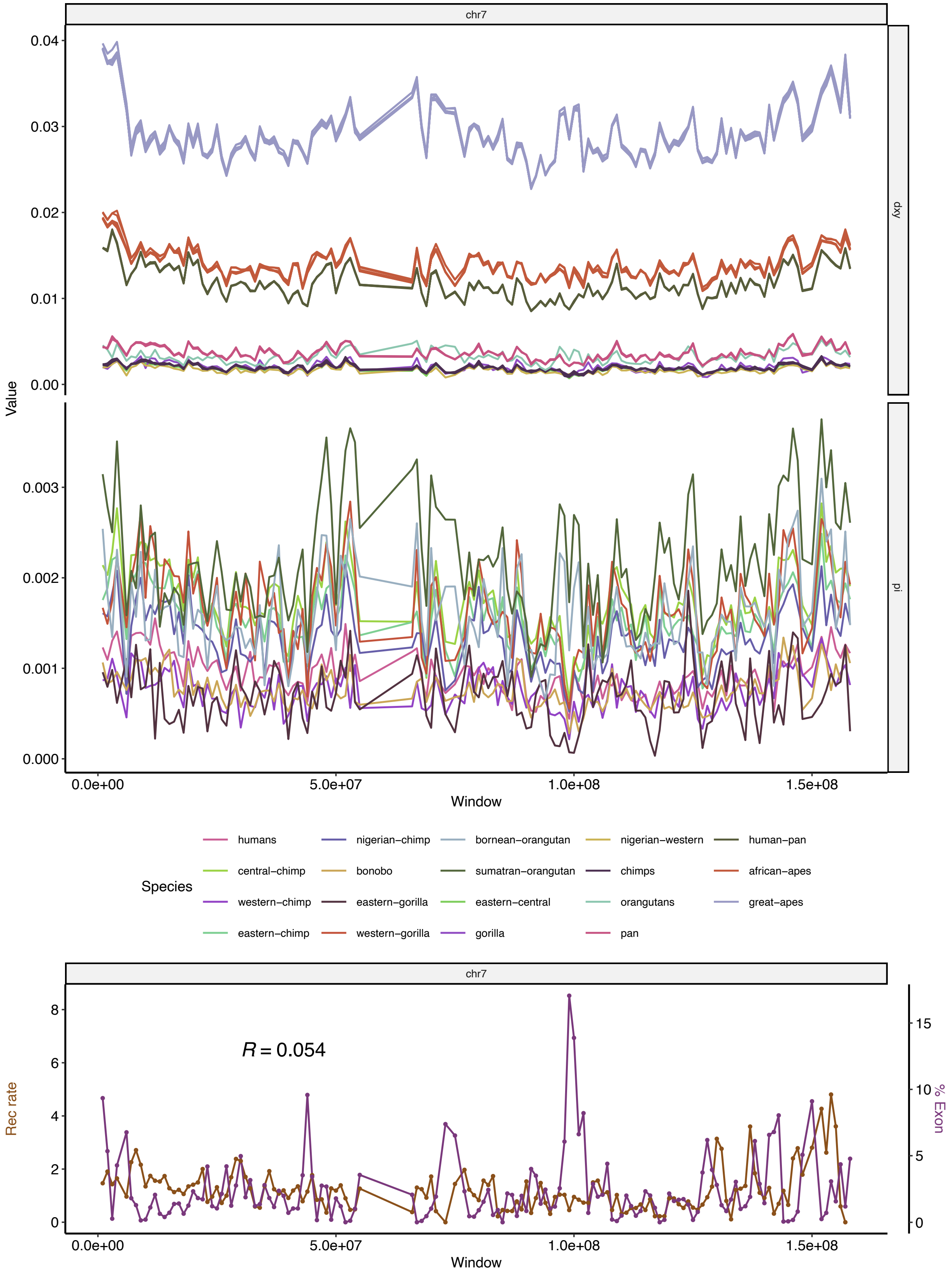

Figure S16: Landscapes of diversity, divergence, exon density and recombination rate across chromosome 7. See Figure 2 for more details.

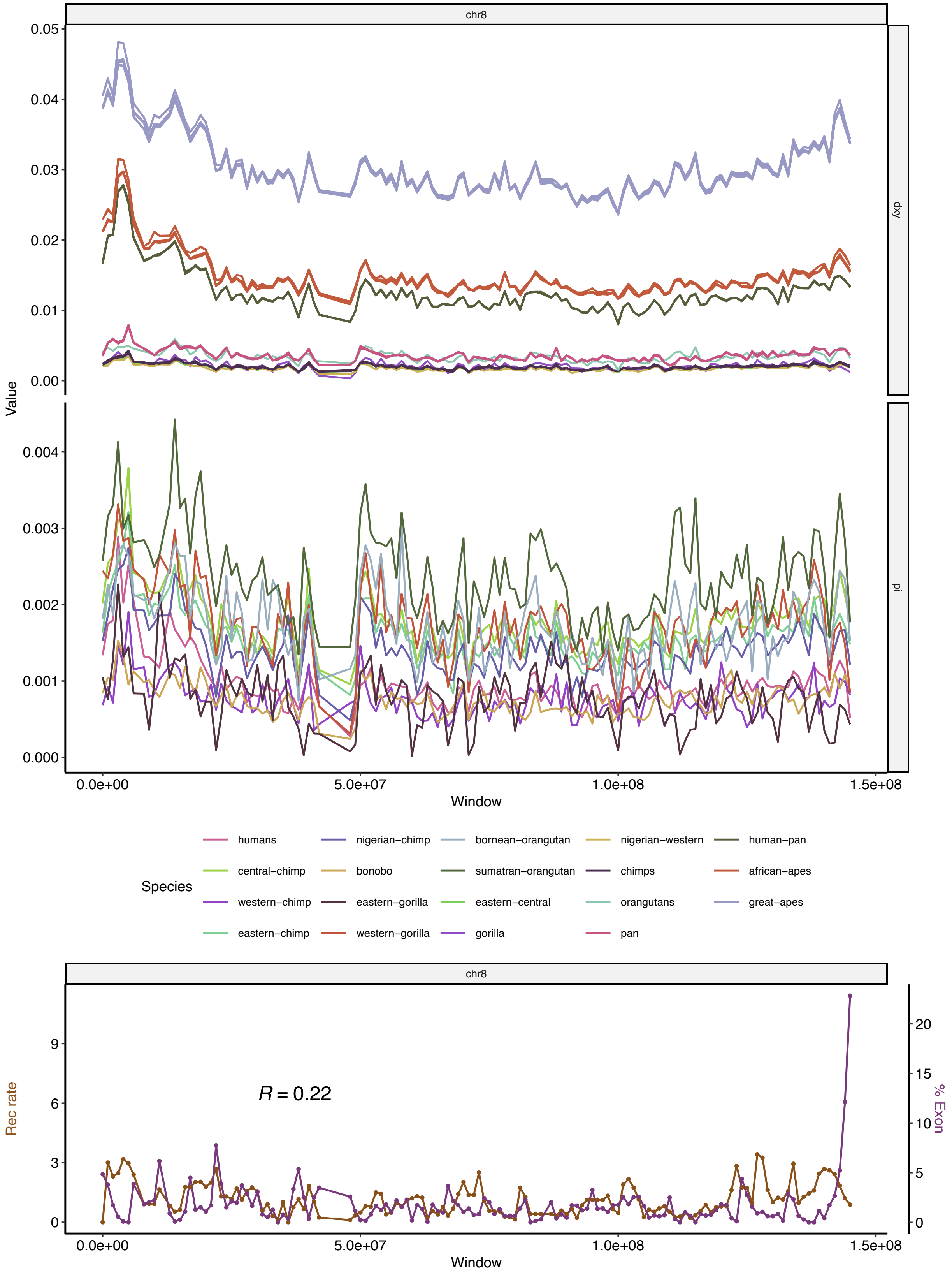

Figure S17: Landscapes of diversity, divergence, exon density and recombination rate across chromosome 8. See Figure 2 for more details.

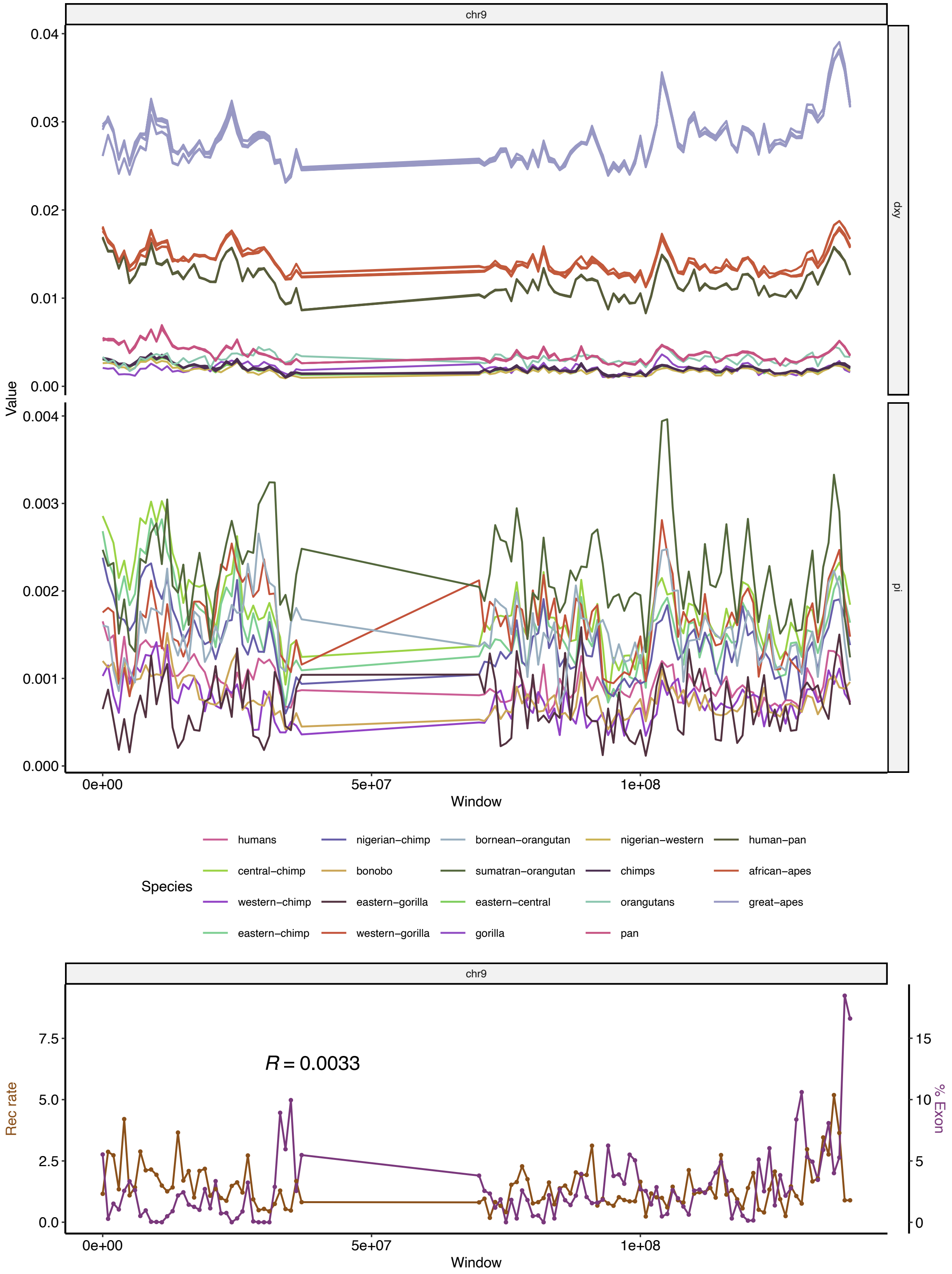

Figure S18: Landscapes of diversity, divergence, exon density and recombination rate across chromosome 9. See Figure 2 for more details.

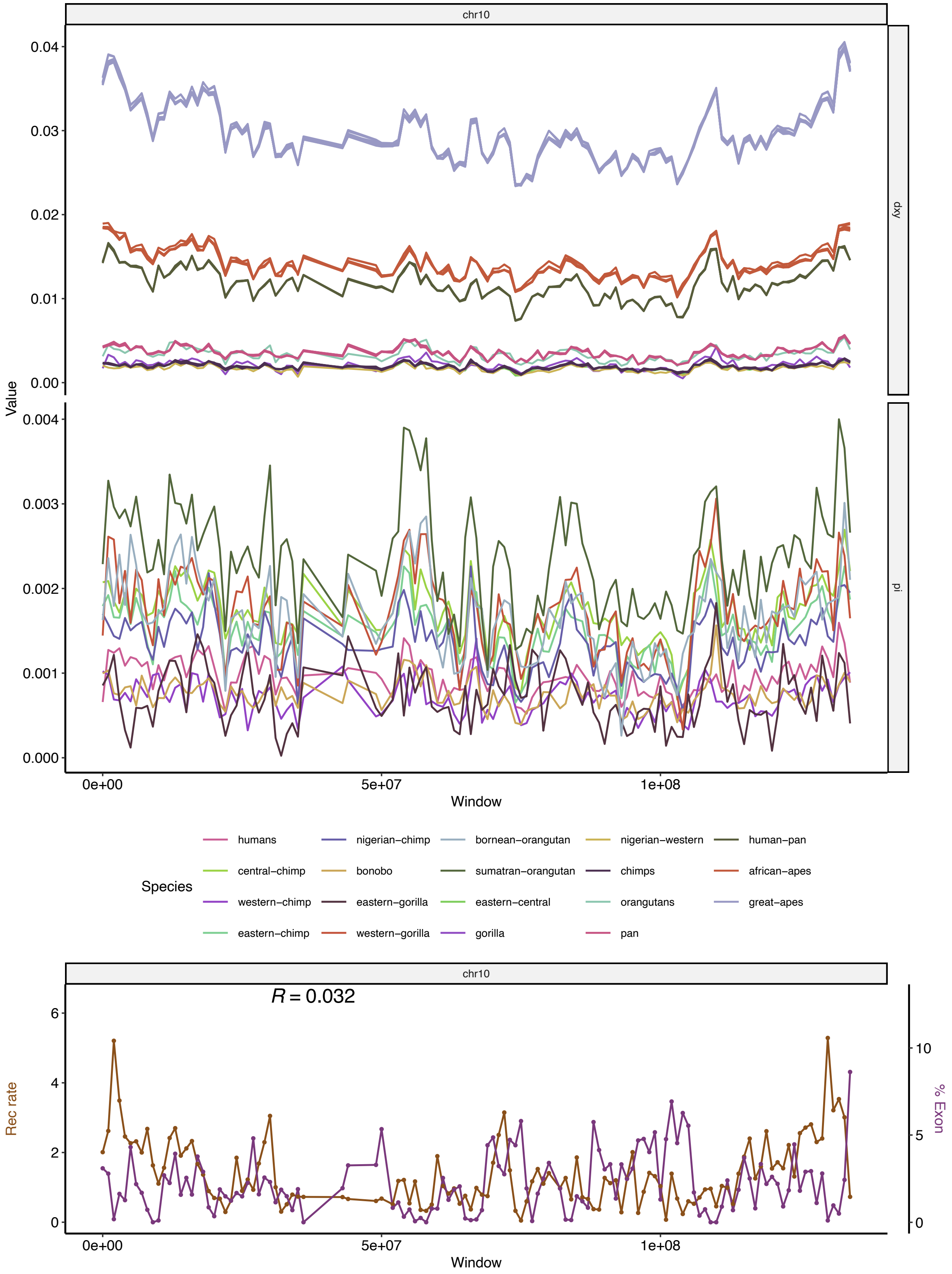

Figure S19: Landscapes of diversity, divergence, exon density and recombination rate across chromosome 10. See Figure 2 for more details.

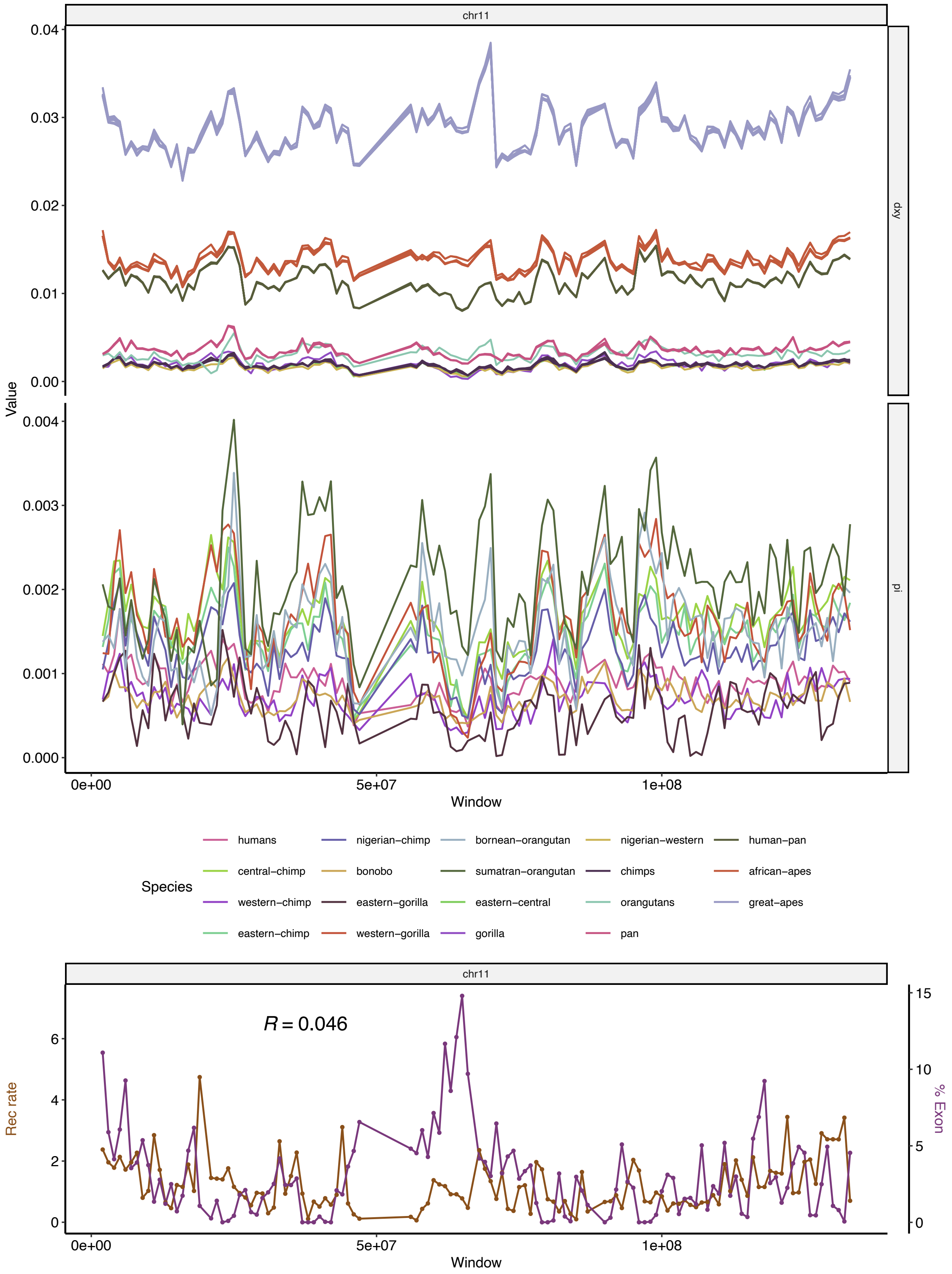

Figure S20: Landscapes of diversity, divergence, exon density and recombination rate across chromosome 11. See Figure 2 for more details.

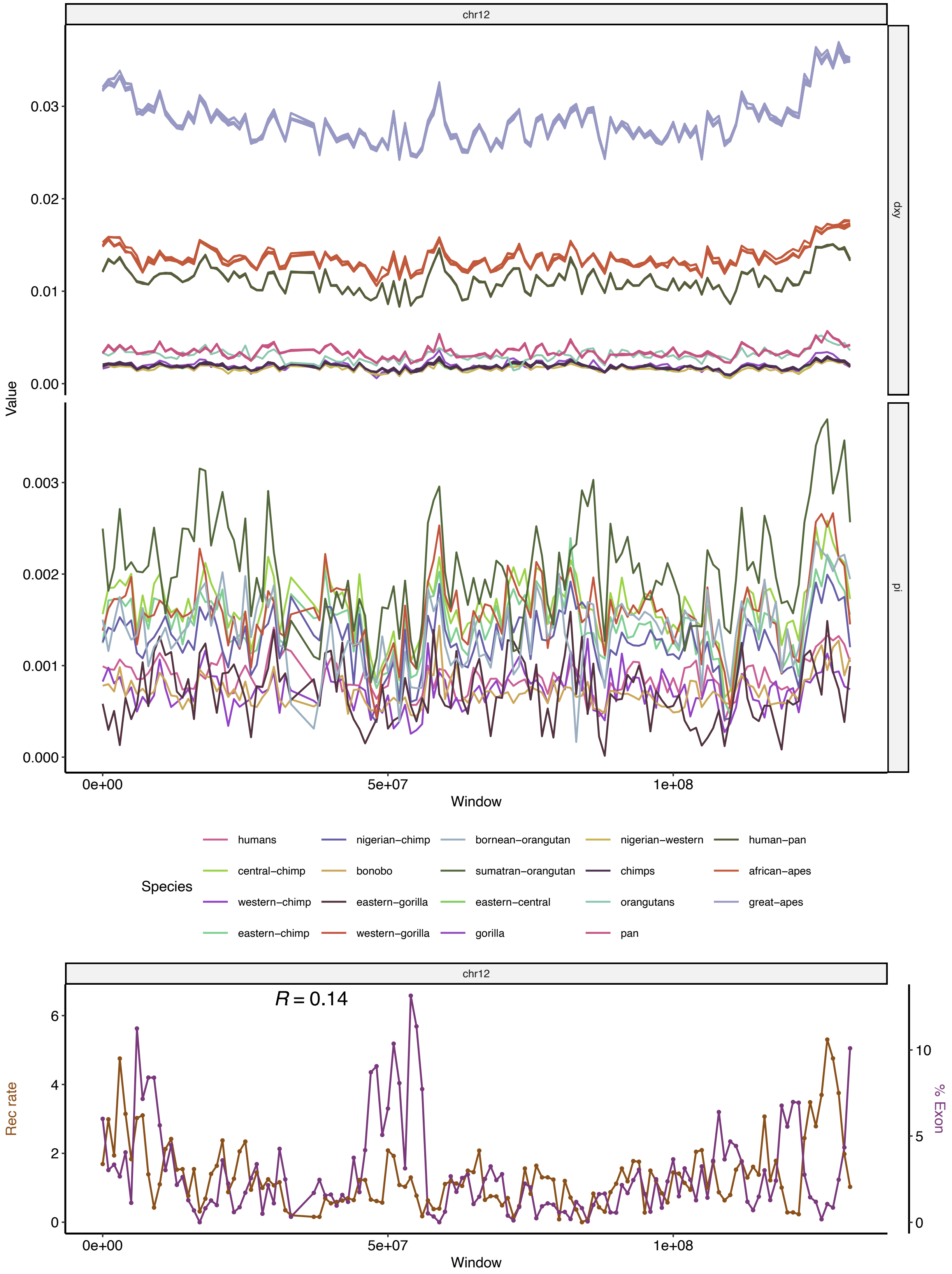

Figure S21: Landscapes of diversity, divergence, exon density and recombination rate across chromosome 12. See Figure 2 for more details.

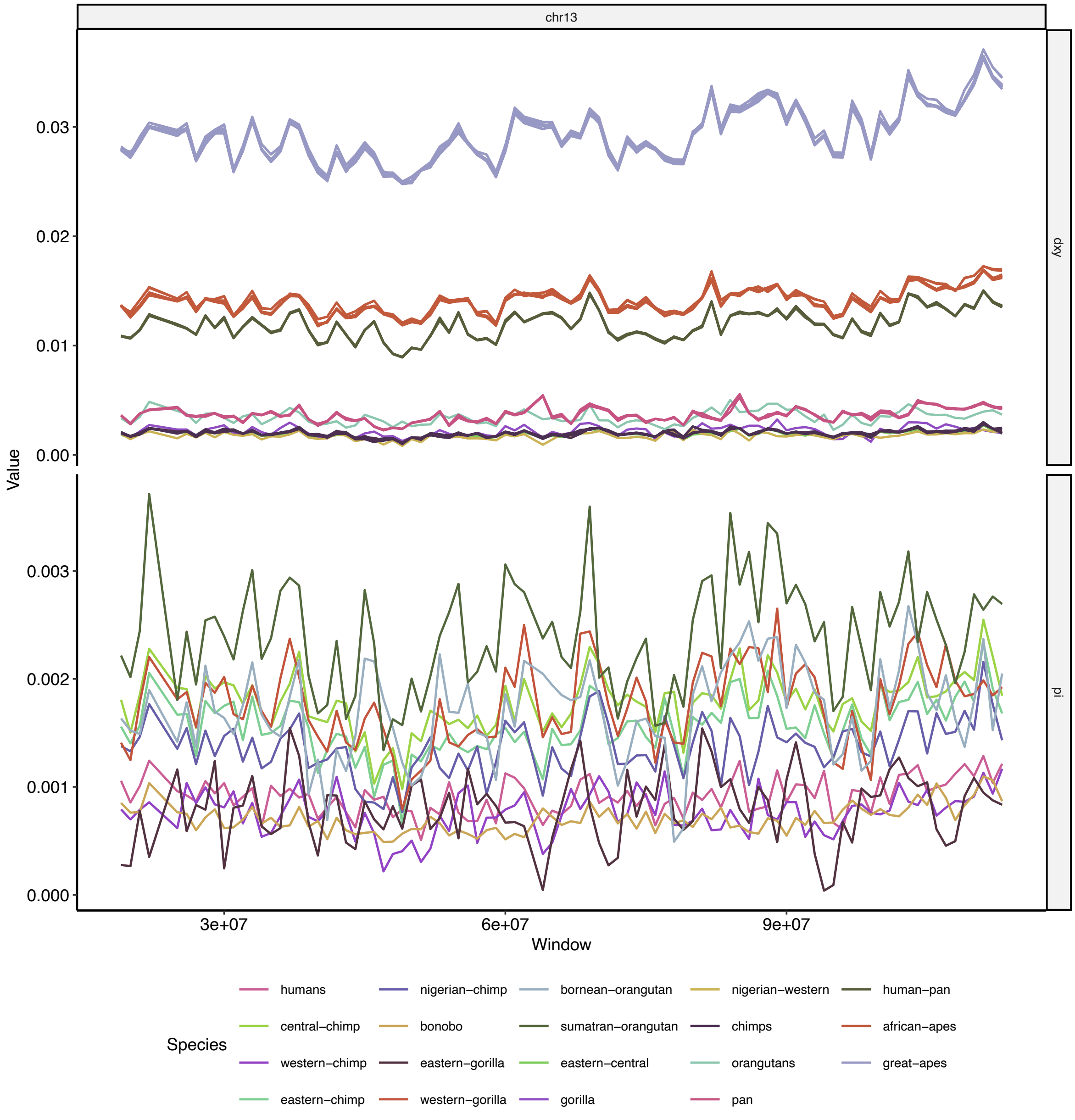

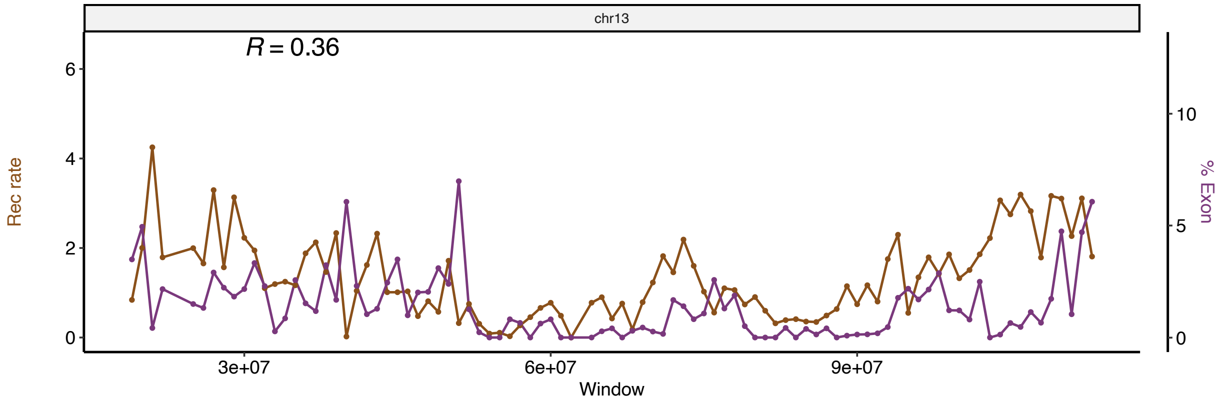

Figure S22: Landscapes of diversity, divergence, exon density and recombination rate across chromosome 13. See Figure 2 for more details.

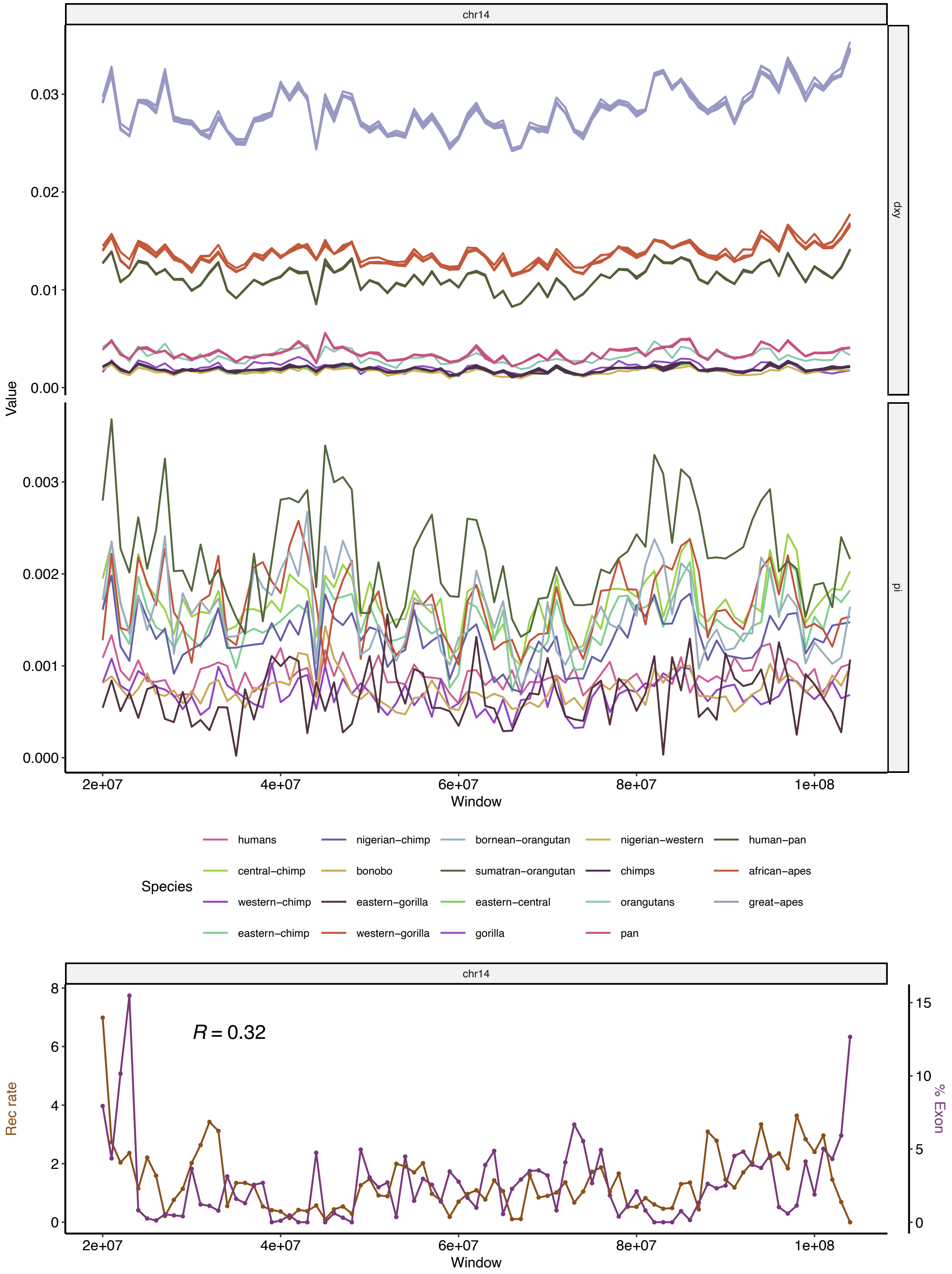

Figure S23: Landscapes of diversity, divergence, exon density and recombination rate across chromosome 14. See Figure 2 for more details.

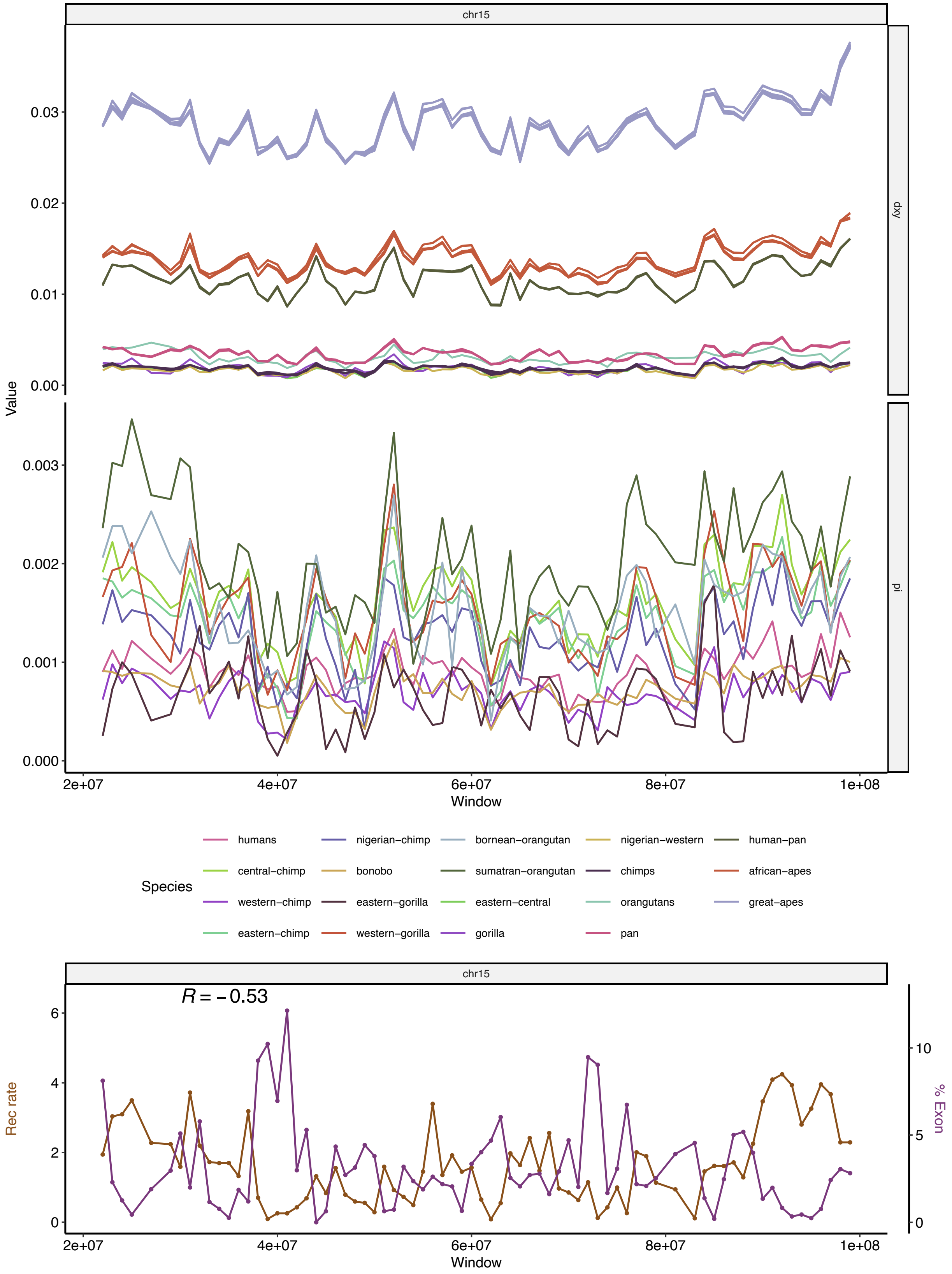

Figure S24: Landscapes of diversity, divergence, exon density and recombination rate across chromosome 15. See Figure 2 for more details.

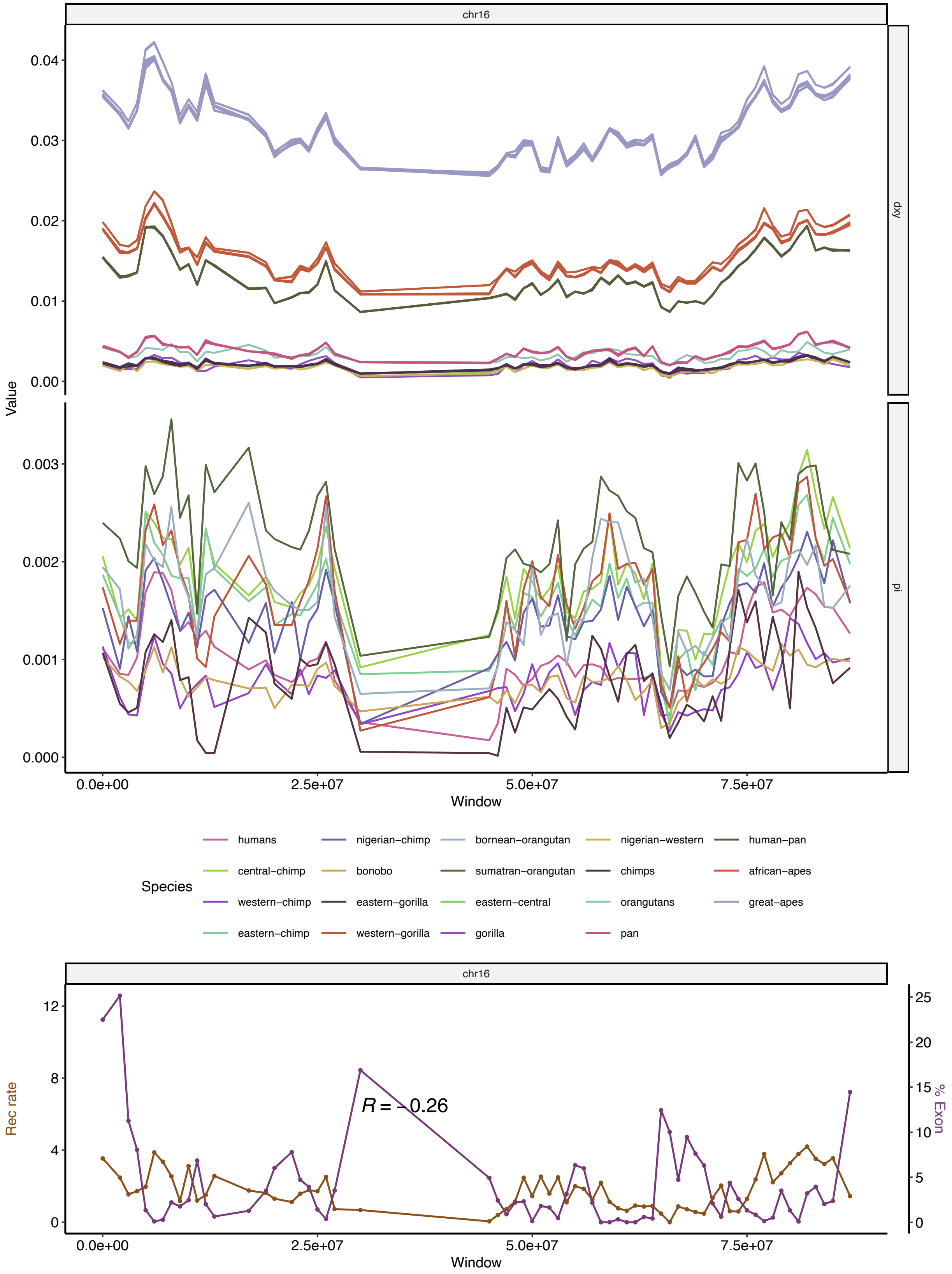

Figure S25: Landscapes of diversity, divergence, exon density and recombination rate across chromosome 16. See Figure 2 for more details.

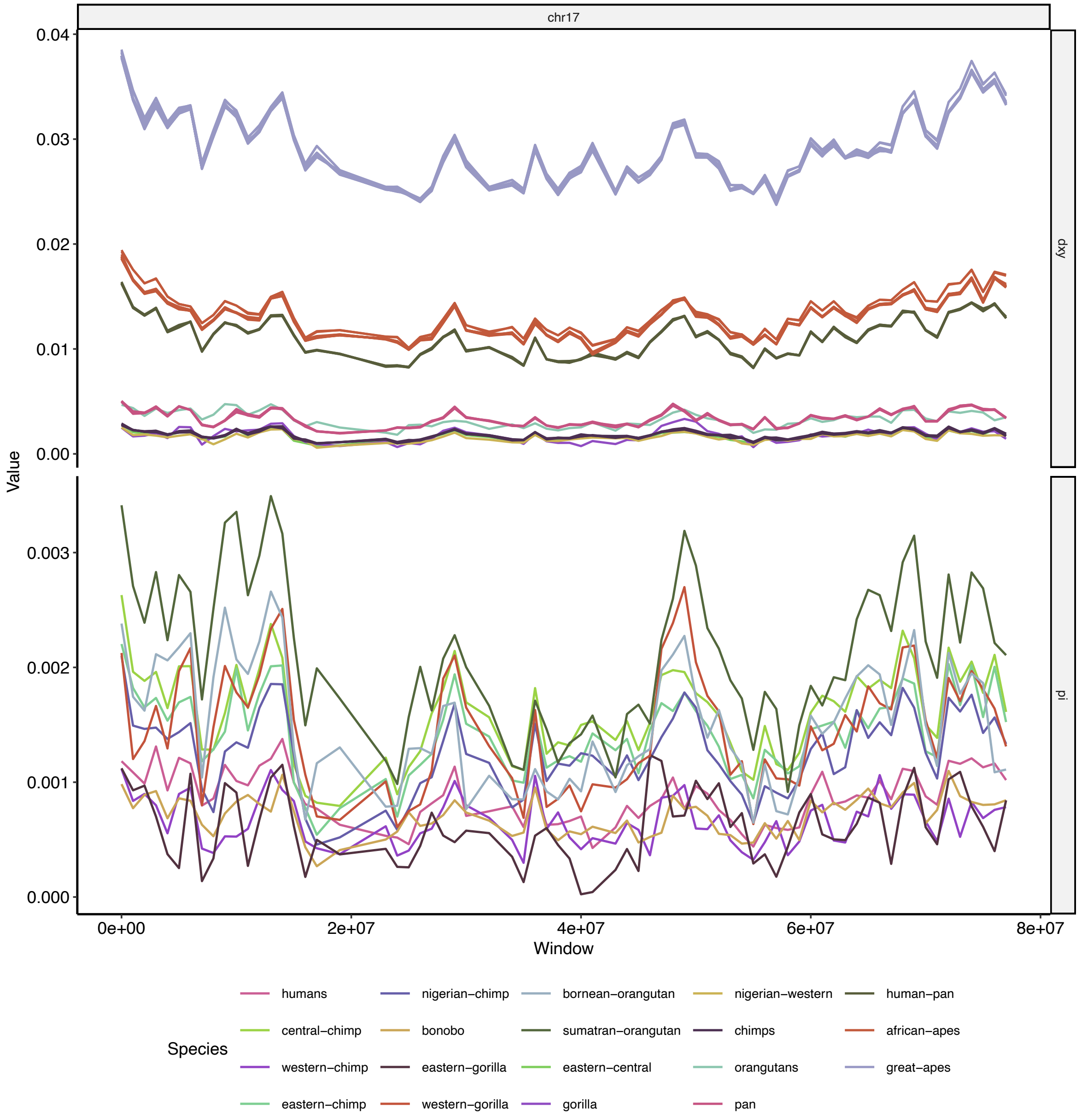

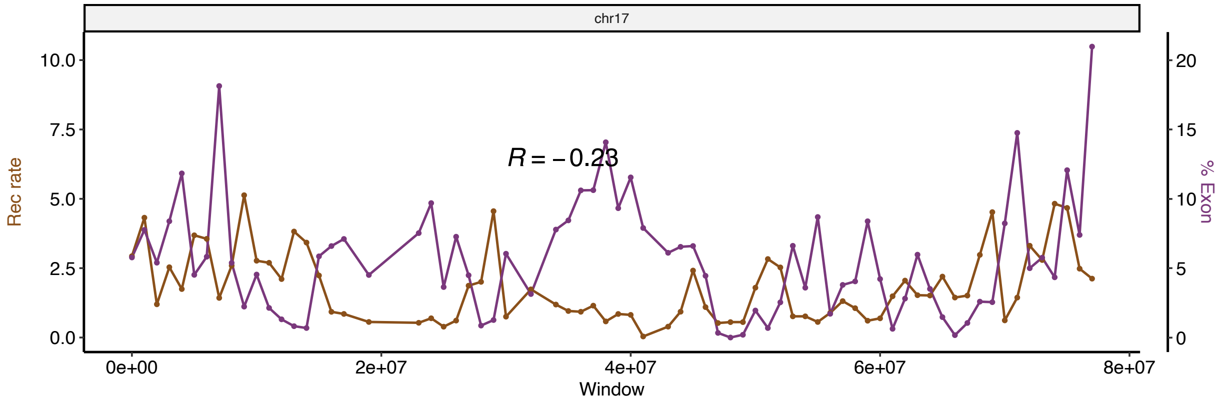

Figure S26: Landscapes of diversity, divergence, exon density and recombination rate across chromosome 17. See Figure 2 for more details.

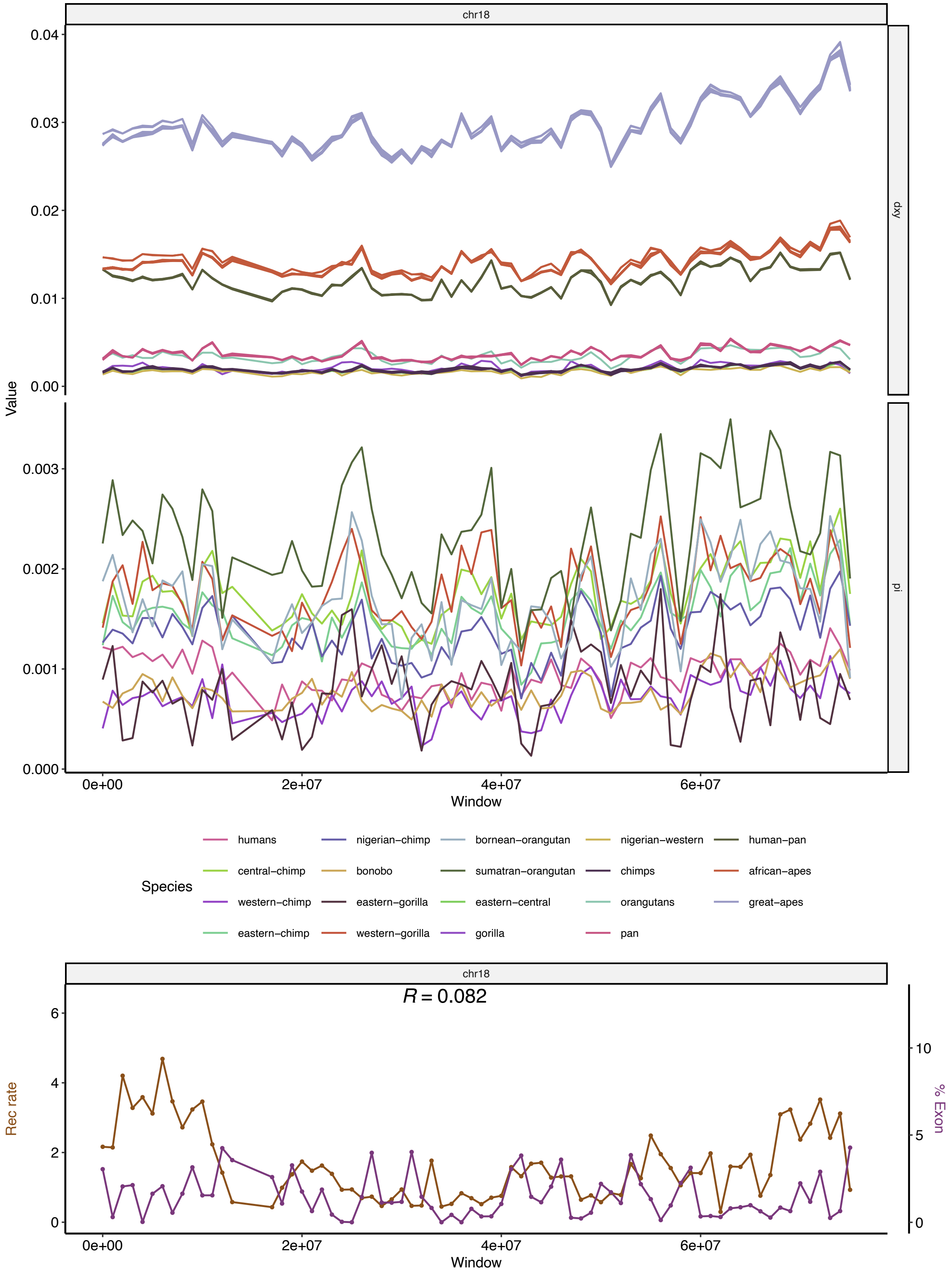

Figure S27: Landscapes of diversity, divergence, exon density and recombination rate across chromosome 18. See Figure 2 for more details.

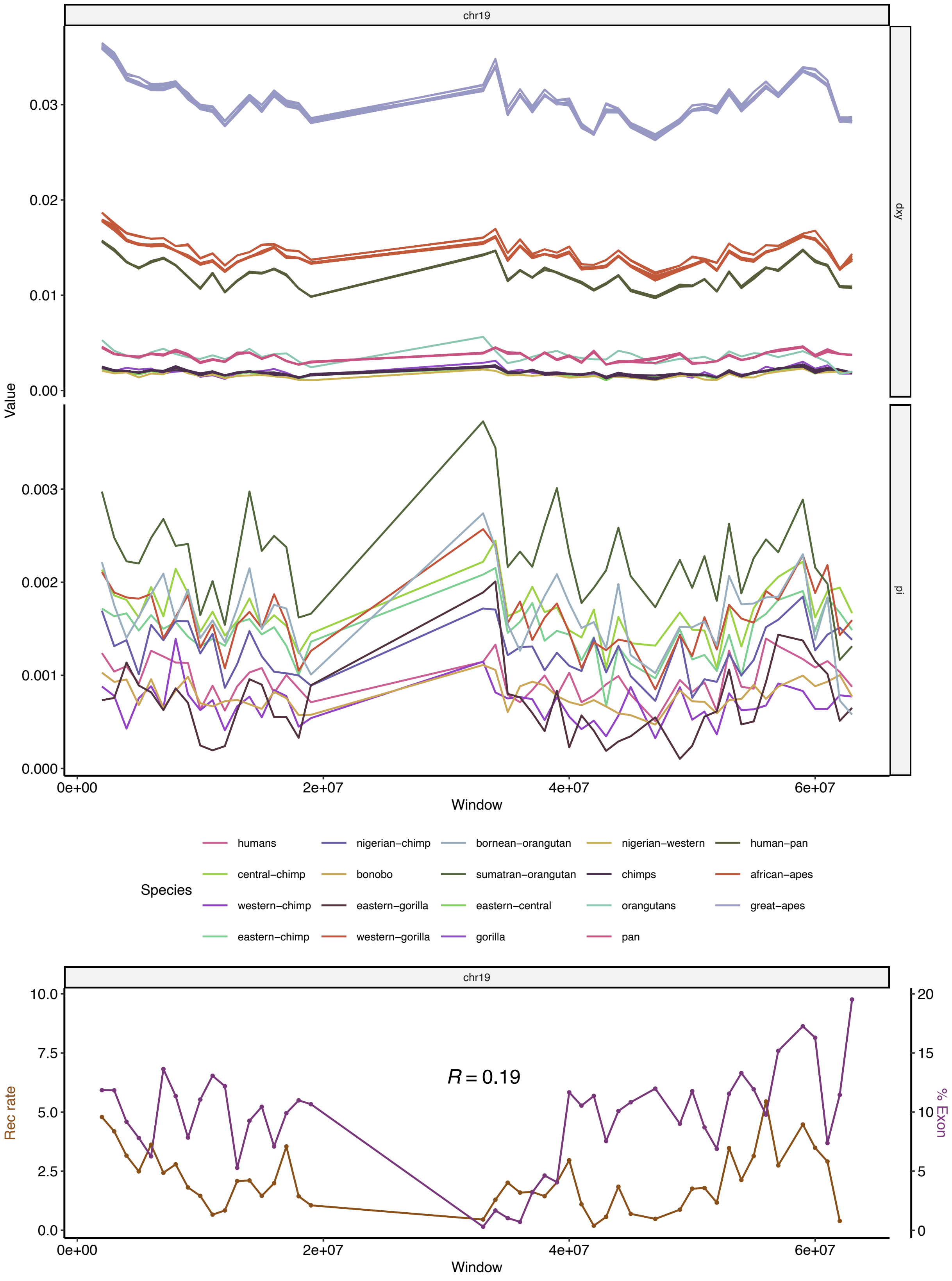

Figure S28: Landscapes of diversity, divergence, exon density and recombination rate across chromosome 19. See Figure 2 for more details.

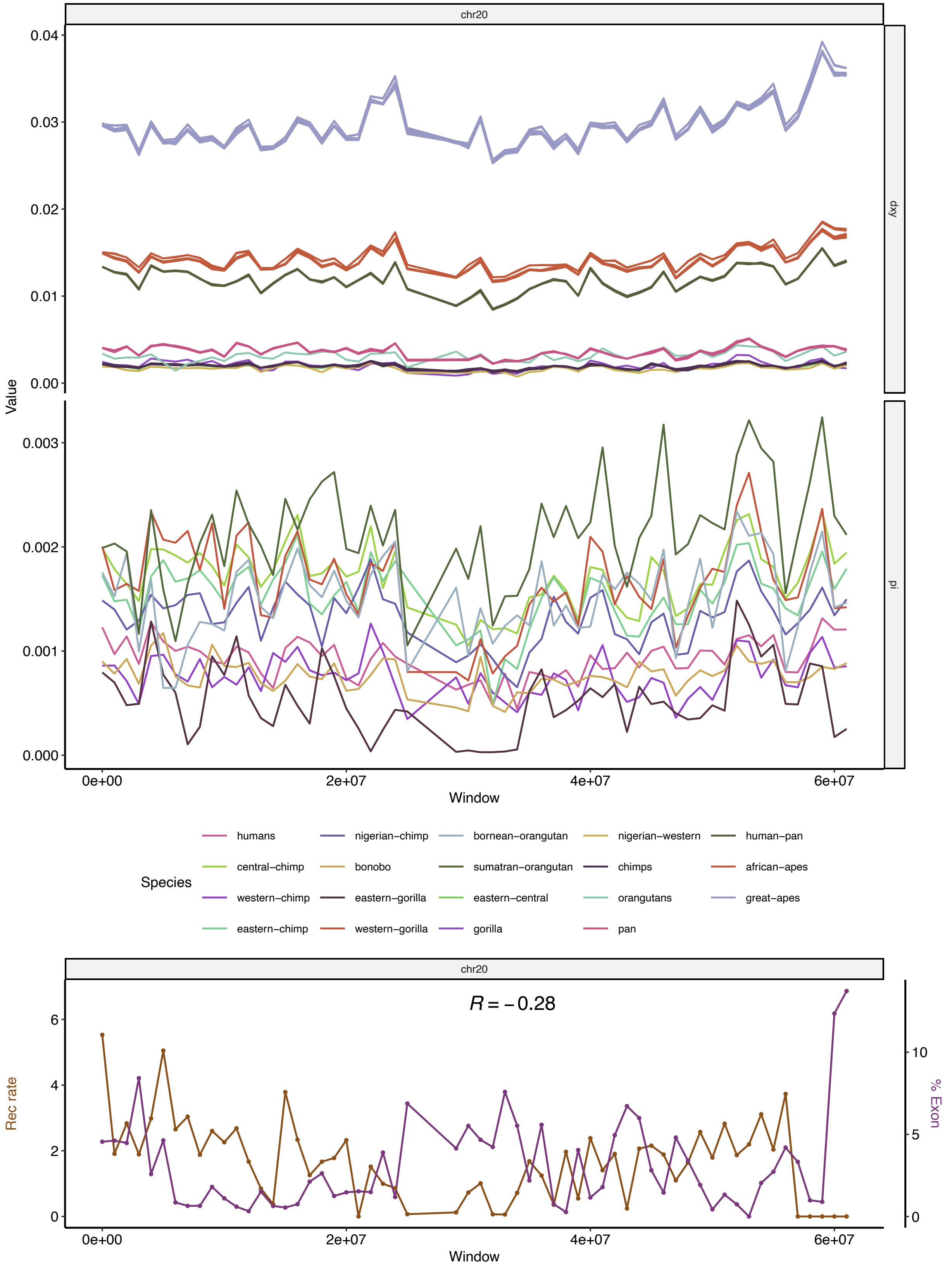

Figure S29: Landscapes of diversity, divergence, exon density and recombination rate across chromosome 20. See Figure 2 for more details.

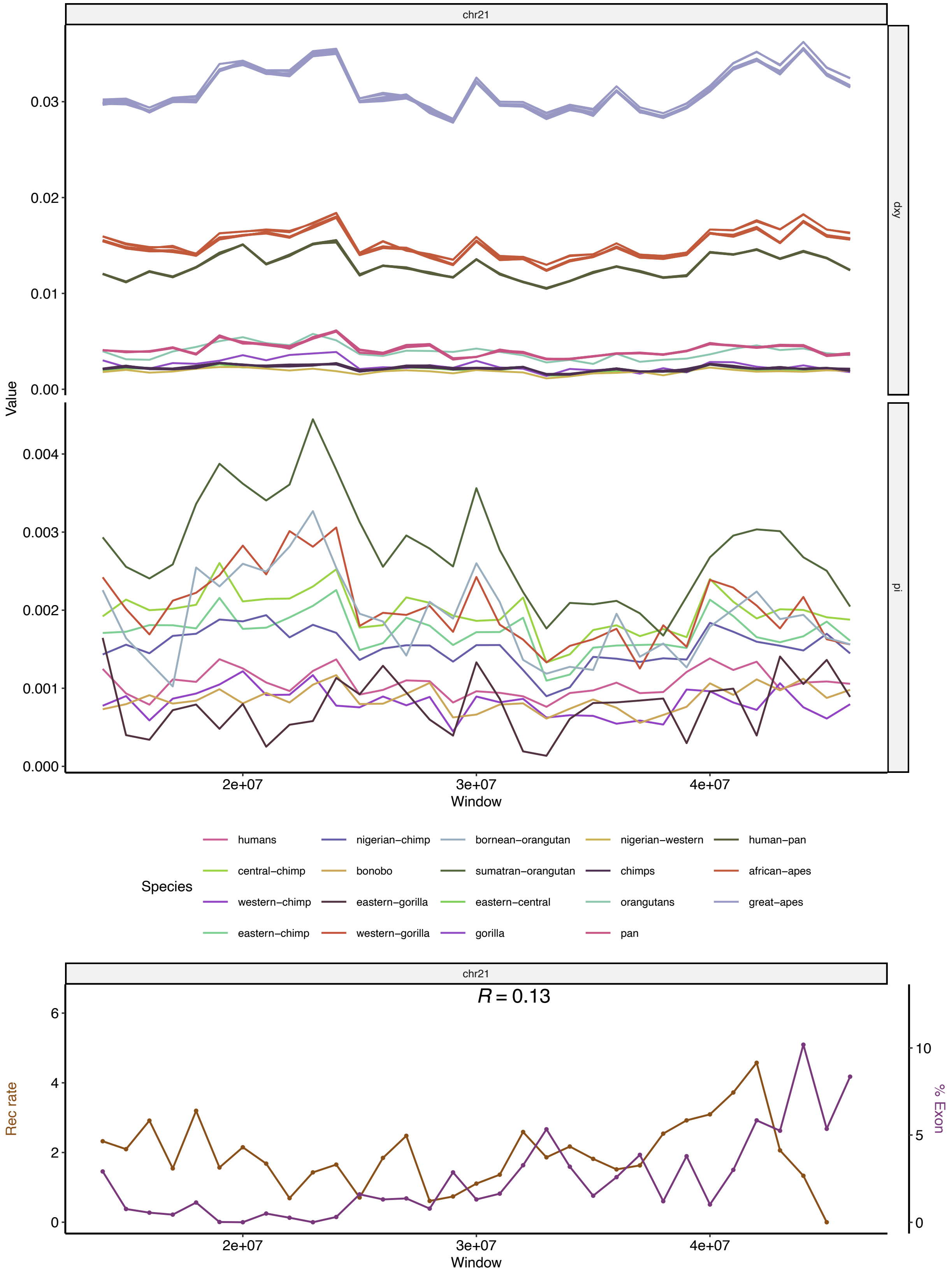

Figure S30: Landscapes of diversity, divergence, exon density and recombination rate across chromosome 21. See Figure 2 for more details.

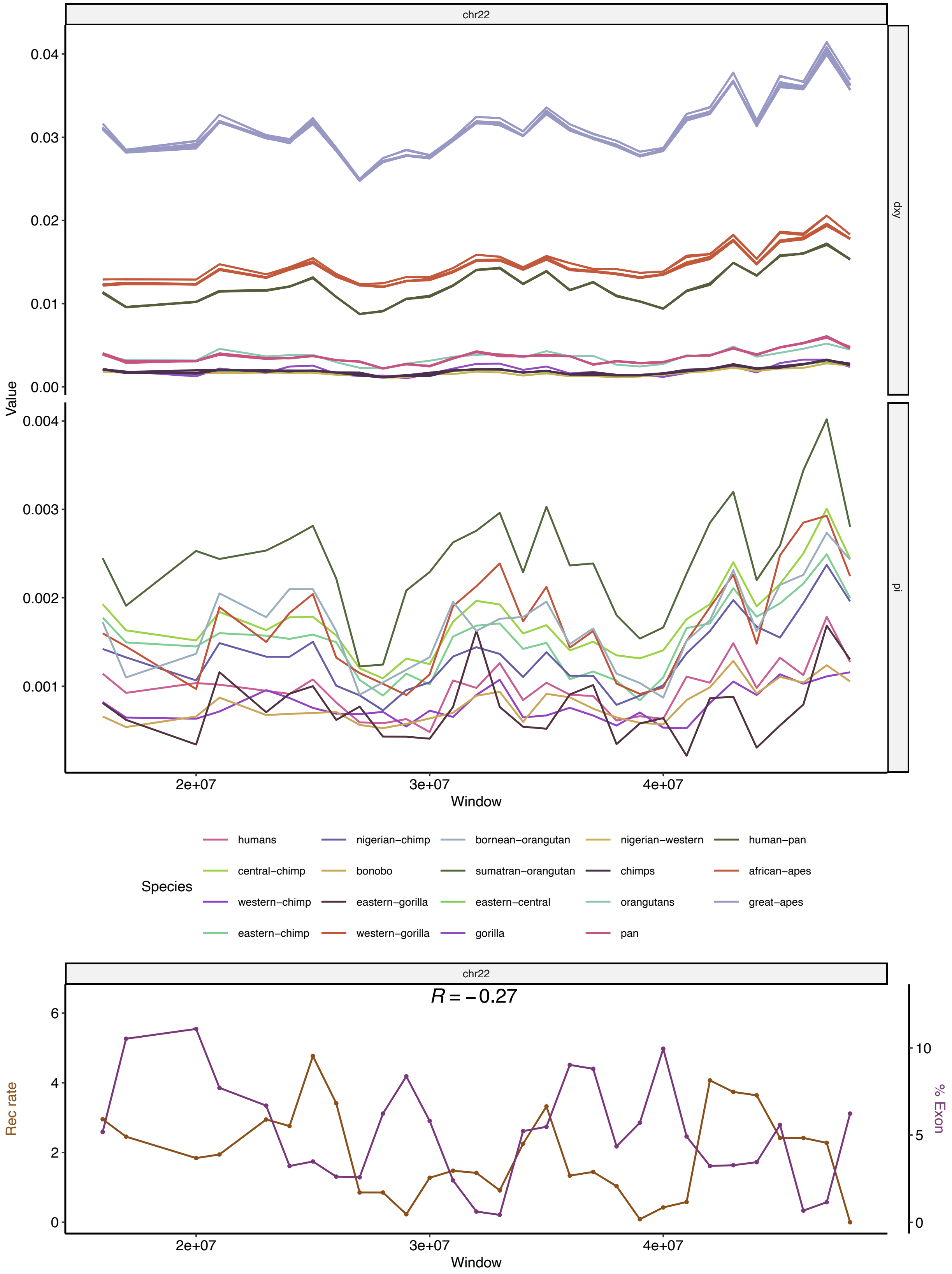

Figure S31: Landscapes of diversity, divergence, exon density and recombination rate across chromosome 22. See Figure 2 for more details.
